# Synthesis of Sulfonamides in the Biobased Solvent Cyrene

**DOI:** 10.1002/open.70290

**Published:** 2026-08-27

**Authors:** Michela Galli, Andrea Fulminante, Alessandro Fiori, Valerio Fasano, Alessandra Silvani, Daniele Passarella, Andrea Citarella

**Affiliations:** ^1^ Department of Chemistry University of Milan Milano Italy

**Keywords:** Cyrene, mild reaction conditions, solvent effect, solvent recyclability, sulfonamides, sulfonyl chloride‐amine coupling, sustainable reaction medium

## Abstract

A versatile protocol for the synthesis of sulfonamides using the biobased solvent Cyrene as reaction medium is reported. A wide range of primary and secondary amines were successfully employed, demonstrating the broad applicability of the method. The reactions proceed at room temperature and typically reach completion within 30–60 min. The resulting 20 sulfonamide products were isolated in good to high yields (up to 90%) by simple precipitation, upon addition of cold water and mild acid, without the need for chromatographic separation. The identified procedure offers an improved environmental profile, while maintaining high efficiency. Cyrene can be recovered and reused without loss of efficiency: Through a two‐step extraction and filtration process, up to 79% of the solvent was successfully recovered and reused in a subsequent reaction cycle, furnishing the product in a yield comparable to that of the first cycle. Overall, this methodology provides a practical, mild, scalable, and more sustainable approach to sulfonamide synthesis.

## Graphical abstract

An alternative and efficient protocol for sulfonamide synthesis using the biobased solvent Cyrene is reported. A broad range of amines reacts smoothly with sulfonyl chlorides at room temperature, furnishing 20 sulfonamides in moderate to high yields within 30–60 min. Products are isolated by simple precipitation without chromatography, while Cyrene can be recovered (up to 79%) and reused without loss of performance.

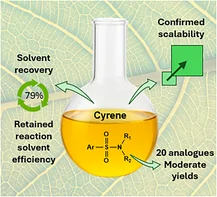

## 1 Introduction

**FIGURE 1:**
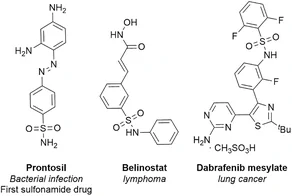
FDA‐approved drugs based on the sulfonamide scaffold [2].

Sulfonamides are organosulfur compounds characterized by the presence of the ─SO_2_–NR_1_R_2_ functional group: This scaffold started to be investigated in the early 20th century for their antimicrobial activity, with the discovery of the first sulfonamide drug, Prontosil, in the 1930s. Since then, the sulfonamide scaffold has been widely studied for its biological activity, leading to numerous FDA‐approved sulfonamide drugs used as diuretic, antidiabetic, hypoglycemic, antiviral, or anticancer agents, or protease inhibitors [1] (Figure 1).

Due to the high pharmaceutical interest of this class of compounds, considerable efforts have been devoted to the development and optimization of synthetic protocols for the preparation of sulfonamides. Despite the availability of numerous methodologies, the reaction between sulfonyl chlorides and amines remains the method of choice as it is simple and the required starting materials are easily available. However, the classical procedure often requires the use of harsh reaction conditions for poorly nucleophilic amines, the formation of undesired byproducts, including over‐sulfonylation of primary amines and, in addition, the use of toxic solvents such as dichloromethane (DCM), dimethylformamide (DMF), or N‐methyl pyrrolidone (NMP). To overcome these limitations, extensive research over the last decades has focused mainly on two strategies: the introduction of catalysts to improve the reaction outcome and the identification of alternative reaction solvents. In this context, Meshram et al. [3] reported a base‐free synthesis of sulfonamides, using cupric oxide as catalyst in acetonitrile at room temperature, affording a variety of sulfonamides in good yields (70%–92%) within reaction times ranging from 1 to 8 h. Other metals have also been investigated; for instance, Kim et al. [4] described the use of indium, in place of copper oxide, in acetonitrile: In this case, indium proved to be less efficient than copper, providing 13 examples of differently substituted sulfonamides in good yields (71%–90%), at room temperature within 6–16 h. A more specialized and exotic approach was reported by Rattanburi et al. [5], who employed magnetic nanoparticle‐supported *N*,*N*‐diisopropylaminoacetamide (Fe_3_O_4_‐DIPA) as magnetic catalyst: The supported base in this way can be easily removed by magnetic separation. With this approach, the reaction goes in short times at room temperature, even if a limited number of examples are reported. Nevertheless, considering the broad pharmaceutical applicability of sulfonamides, the avoidance of metal catalysts is often desirable. An alternative approach has therefore focused on the use of reaction media capable of enhancing substrate reactivity while offering improved environmental profiles. Jyoti Das et al. [6] reported for example the synthesis of sulfonamides in PEG‐400 together with K_2_CO_3_ in a heterogeneous system. Although polyethylene glycol exhibits reduced toxicity and flammability compared to conventional organic solvents, the protocol still required elevated temperatures (60–120 °C), thus not fully addressing the issue of harsh reaction conditions. Solvent‐free approaches have also been explored; for example, Alavi et al. [7] reported the synthesis of quinoxaline‐based sulfonamides under neat, catalyst‐free conditions using the amine as both reagent and solvent in the presence of NaHCO_3_. Despite its novelty, this method is limited to liquid aromatic amines. More promising results were obtained under aqueous conditions: Deng and Mani [8] proposed an alternative protocol in which the sulfonyl chloride and the amine were suspended in water with potassium carbonate, affording sulfonamides in moderate to excellent yields in short times. However, this methodology was limited by the need of water‐soluble substrates and strict pH control. Water, sometimes combined with acetonitrile, has also been employed in electrochemical [9], photochemical [10], or flow‐chemistry [11] approaches, which generally provided excellent results but remained constrained by substrate solubility. In light of the ongoing search for efficient, versatile, and mild conditions for the synthesis of sulfonamides, we investigated the use of Cyrene as reaction solvent. Cyrene (dihydrolevoglucosenone) is a bicylic acetal obtained from cellulose through pyrolysis followed by hydrogenation [12]. This solvent presents high solvent power, high boiling point at both ambient pressure and vacuum conditions (b.p. = 222 °C at 1013 mbar, 100 °C at 10 mbar [13]), high polarity, and a good miscibility with water. Moreover, Cyrene is biodegradable, nonmutagenic, and nontoxic (LD_50‐oral_ > 2000 mg/kg [13]) making it a sustainable alternative to conventional organic solvents [14]; Cyrene has found in recent years application in several organic reactions, such as azide–alkyne click cycloadditions [15] and nucleophilic aromatic substitutions [16], as well as in several kinds of C─C cross coupling reactions [17, 18]. These works demonstrated that Cyrene often reduces reaction times; considering its appealing chemical and physicochemical properties, the applicability of Cyrene to sulfonamide synthesis was evaluated. Pleasingly, we developed a simple, mild, and rapid protocol that enabled the preparation of a library of 20 sulfonamides from sulfonyl chlorides and a wide range of amines. Compared to existing methodologies, the use of Cyrene allows to perform reactions at room temperature and applicable to both water‐soluble and water‐insoluble substrates. The reaction reaches completion in reduced time (30–60 min) and allows to recover the final product through simple precipitation upon addition of an aqueous acidic solution followed by iced water. It is to note that despite the presence of bases in the reaction mixtures, no polymerization of Cyrene [19] has been observed in any of the cases reported in this work. The use of organic bases is in fact compatible with the use of Cyrene without any polymerization side effects. For what concerns inorganic bases, Cyrene polymerization is usually observed with harsher reaction conditions, stronger inorganic bases, higher reaction temperatures, or longer reaction times. In our case, the short reaction time (30–60 min) and the low temperature (rt) allow to recover the product in good yield without observing any side reactivity.

**FIGURE 2:**
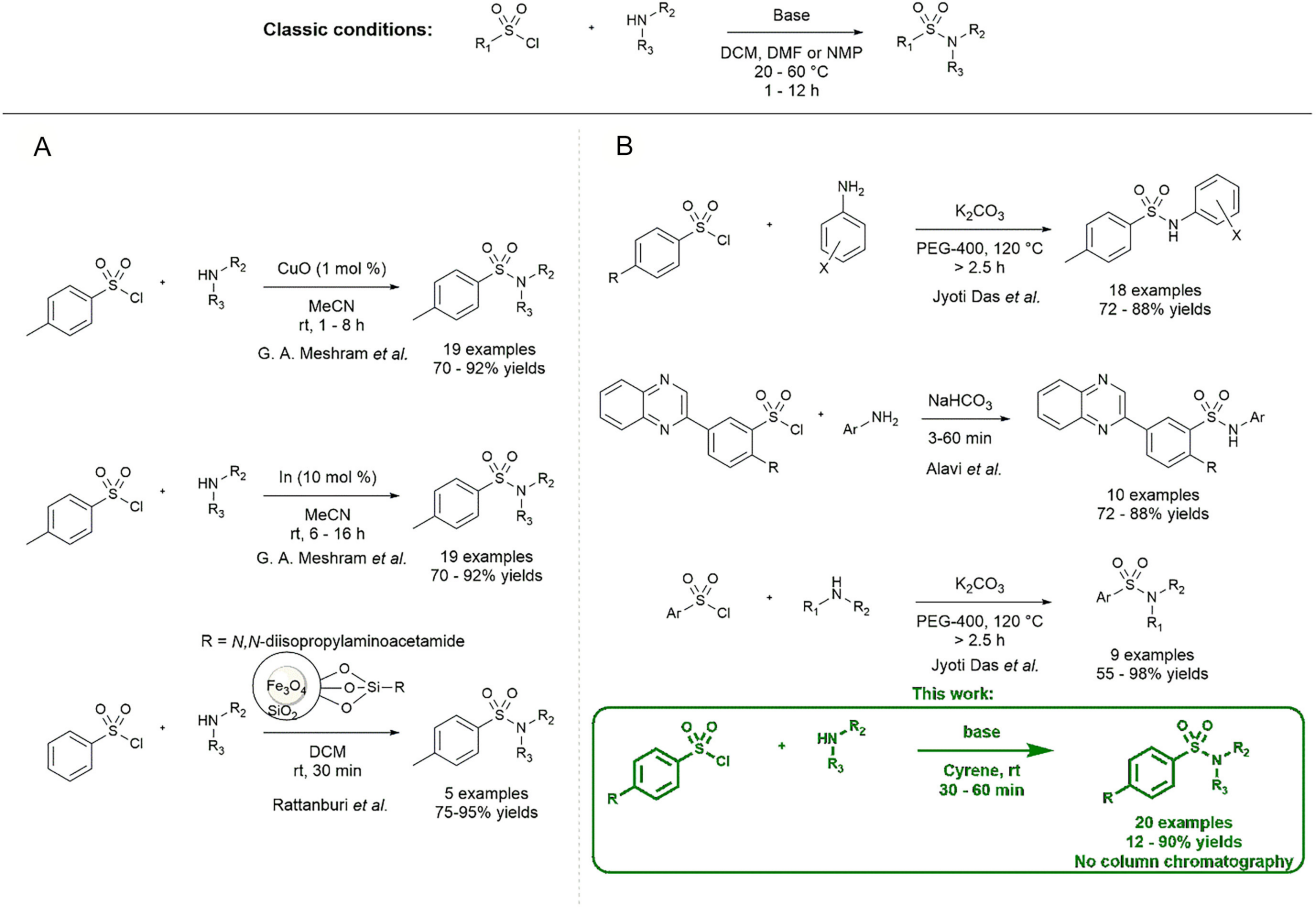
Methodologies currently employed for the synthesis of sulfonamides: (A) methodologies including the presence of a catalyst and (B) methodologies performed in alternative reaction solvents.

## 2 Results and Discussion

### 2.1 Reaction Scope

The formation of sulfonamides in Cyrene was initially attempted using *p‐*toluene sulfonyl chloride (TsCl) and benzylamine in the presence of the inorganic base NaHCO_3_ (Scheme ‍1). The two starting materials were simply dissolved in Cyrene, the base was added, and the reaction mixture was left under vigorous stirring (necessary due to the high viscosity of Cyrene) for the appropriate reaction time. After the full conversion of the starting material, as observed by TLC analysis, the reaction flask was cooled to 0 °C and HCl 1 M was added until pH ≈ 3–4. After the addition of iced water, precipitation of the product was observed: The obtained solid was recovered through filtration and washed with water to remove the excess of Cyrene, allowing to obtain the pure desired product **1** in 79% yield without the need for further purification. The same procedure was followed using 4‐methoxybenzylamine, and similar satisfying results were obtained (**2**, 81% yield). The reaction was attempted also with 2‐aminomethylpyridine as amine component: In this case, the reaction did not proceed with an inorganic base, and therefore, the use of triethylamine (TEA) was necessary to obtain the desired sulfonamide (**3**, 31% yield). The presence of the pyridine ring required moreover a precise pH control: An excessively acid pH led in fact to the formation of water‐soluble pyridinium salts. For this reason, a milder aqueous solution of NaHSO_4_ 0.5 M was used in place of HCl 1 M, and iced water was added when neutral pH was reached.

**SCHEME 1:**
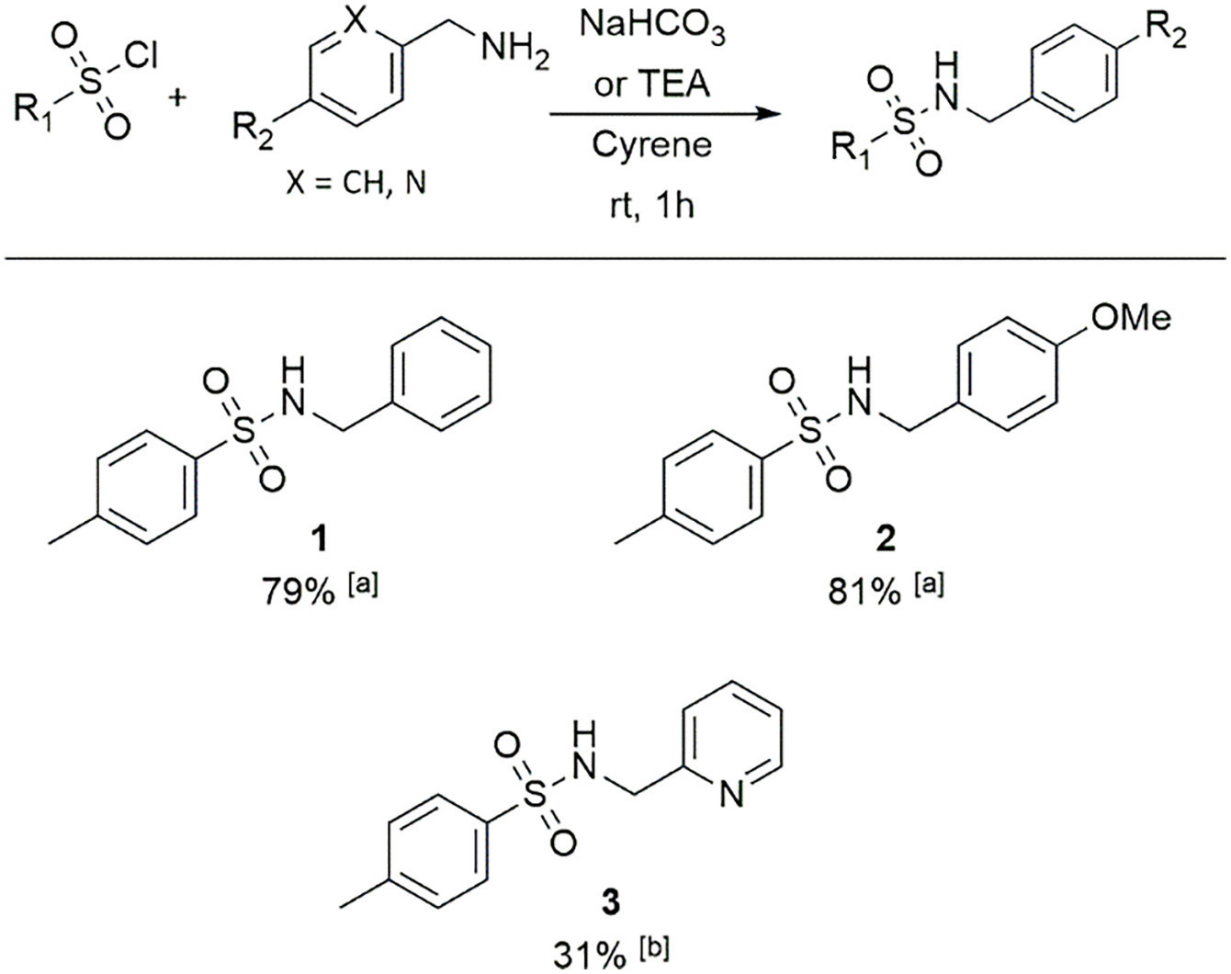
Synthesis of sulfonamides **1**–**3**. Reagents and conditions: TsCl (1.0 mmol, 0.25 M), appropriate amine (1.0 mmol), NaHCO_3_ (1.5 mmol) or TEA (2.0 mmol), and Cyrene (4 mL) at room temperature for 1 h. ^[^^a^^]^NaHCO_3_ was used as base; ^[^^b^^]^TEA was used as base.

We then switched to the investigation of anilines’ reactivity: 4‐Bromoaniline was initially reacted with *p*‐toluene sulfonyl chloride in the presence of the inorganic base NaHCO_3_, but no product formation was observed (Table 1, entry 1). In the same way, the use of K_2_CO_3_, did not lead to the formation of the product, even if the reaction was left stirring overnight (entry 2). Switching to organic bases, the use of TEA led to the formation of the desired product **A**, together with the undesired side product **B** in a 1:2 ratio (entry 3). The same results were obtained using DIPEA (entry 4). The use of pyridine as a base finally allowed us to obtain selectively the monosubstituted product **A** in good yield (entry 5). The product precipitation was allowed by the addition of an aqueous solution of HCl 1 M until pH ≈ 3–4, followed by iced water.

**TABLE 1:** Optimization of the conditions for the reaction of TsCl and 4‐bromoaniline.^a^

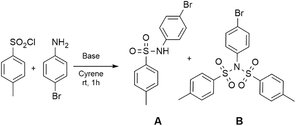			
Entry	Base	*t*	Product
1	NaHCO_3_	30 min	0%
2	K_2_CO_3_	Overnight	0%
3	TEA^b^	1 h	A:B (1:2)^c^
4	DIPEA^b^	3 h	A:B (1:2)^c^
5	Pyridine^d^	30 min	64%^e^

a The reactions have been carried out in a 1 mmol scale, using TsCl (1.0 mmol, *c* = 0.25 M), 4‐bromoaniline (1.0 mmol), and base (1.5 mmol) in Cyrene (4.0 mL) at room temperature for the appropriate time.

b 2.0 mmol of base was used.

c HPLC yield.

d 2.1 mmol of base was used.

e Isolated yield.

**SCHEME 2:**
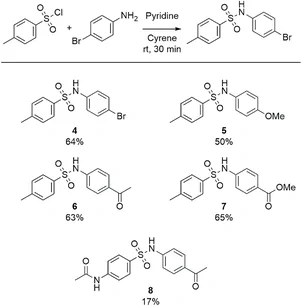
Synthesis of sulfonamides **4**–**8**. Reagents and conditions: TsCl or *p*‐acetamidobenzenesulfonyl chloride (1.0 mmol, 0.25 M), appropriate amine (1.0 mmol), pyridine (2.1 mmol), and Cyrene (4.0 mL) at room temperature for 30 min.

The conditions reported in entry 5 were used for the reaction between TsCl and anilines presenting both electron‐donating and electron‐withdrawing substituents, leading to the desired products in moderate yields (Scheme 2). Also, the reaction with 4‐acetamidobenzenesulfonyl chloride led to the formation of the desired product even if with lower yield (**8**, 17% yield). In all cases, conversion of the sulfonyl chloride was observed after 30 min, so the loss of isolated yield is mainly due to the partial solubility of the products in water.

**SCHEME 3:**
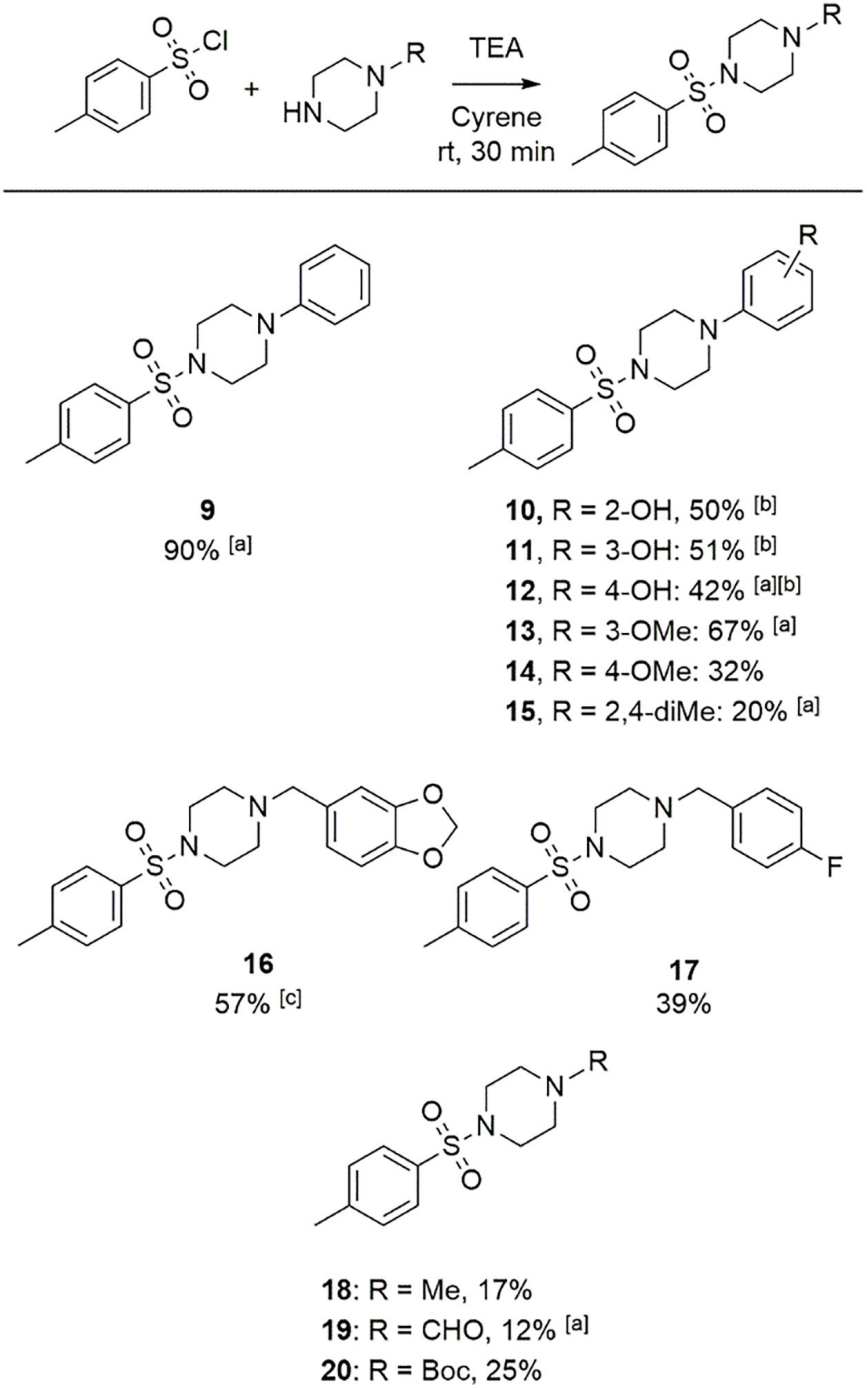
Synthesis of sulfonamides **9**–**20**. Reagents and conditions: TsCl (1.0 mmol, 0.25 M), appropriate *N*‐substituted piperazine (1.0 mmol), TEA (2.0 mmol), and Cyrene (4.0 mL) at room temperature for 30 min. ^[^^a^^]^Reaction time was 1 h; ^[^^b^^]^pyridine (2.0 mmol) was used as base; ^[^^c^^]^reaction time was 2 h.

Eventually, the formation of piperazine‐containing sulfonamides, a class of compounds with increasing biological relevance, was assessed [20, 21]. TsCl was reacted with piperazines bearing phenyl‐, benzyl‐, alkyl‐, or carbonyl‐*N*‐substituents in the presence of triethylamine (Scheme 3). The reaction between TsCl and 1‐phenylpiperazine afforded the desired product in high yield (**9**, 90% yield). When an OH group was present on the aromatic ring of the amine component, a bis‐substituted product was obtained, resulting from the double addition of sulfonyl chloride on both the piperazine NH and the OH group. This outcome is likely due to the basicity of TEA. In fact, switching to pyridine as base allowed the reaction to be selective for the formation of the monosubstituted product (**9**, 50% yield). The influence of the OH substituent in different positions was then investigated, leading to compounds **10** and **11** in moderate yields (51% and 42%, respectively). When the OH group was protected as a methoxy, the desired products could be obtained with the optimized conditions using TEA (**12**, 67% yield; **13**, 32% yield), as well as the product bearing two methyl groups on the phenyl ring (**14**, 20% yield), even if in slightly lower yields probably due to the higher steric hindrance on the sulfonyl chloride moiety. The reaction with *N*‐benzyl substituted piperazines was always successful (**16**, 57% yield; **17**, 39% yield), while *N*‐methyl‐, *N*‐formyl‐, and *N*‐Boc‐piperazines led to the corresponding products in lower yields (**18**, 17% yield; **19**, 12% yield; **20**, 25% yield), probably due to the partial solubility in water.

### 2.2 Scalability

The reaction of TsCl with 1‐phenyl piperazine, which proceeded with 90% yield, was chosen as a model to analyze the scalability of the proposed protocol. Reacting 1.0 g of TsCl (5.58 mmol), the reaction went to completion in 30 min, allowing to obtain the product **9** in a final 87% yield, meaning that increasing the reaction scale does not erode the efficiency of the process.

For the same reaction on 1.0 g of TsCl, classic literature conditions require the use of 1‐phenylpiperazine in the presence of triethylamine, at room temperature overnight. These conditions lead to the formation of the product in 94% yield, which is slightly higher than the one in Cyrene. On the contrary, these conditions require the use of 40 mL of the toxic solvent DCM in place of 13 mL of the nontoxic biobased solvent Cyrene. This slight decrease in yield is therefore compensated by a lower quantity of used reaction solvent. Moreover, the use of DCM does not allow product precipitation, requiring the use of higher quantities of both aqueous and organic solvents to perform the aqueous extraction workup. Last but not least, the use of Cyrene allows to reduce reaction times from overnight. We can therefore conclude that the conditions in Cyrene are actually more convenient than the ones requiring the use of classic organic solvents.

### 2.3 E‐Factor Calculation

**SCHEME 4:**
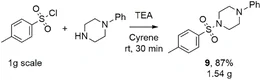
Gram‐scale reaction. Reagents and conditions: TsCl (1 g, 5.58 mmol), 1‐phenyl piperazine (5.58 mmol), TEA (16.7 mmol), and Cyrene (13 mL) at room temperature for 30 min.

Given the promising result obtained from the scale‐up of the reaction, the environmental impact of the proposed protocol was estimated, relying on the E‐factor parameter. The E‐factor gives a quantitative indication of the environmental impact of a protocol: It can be easily calculated by dividing the mass of the waste generated in every phase of the reaction by the mass of the generated product. The gram‐scale reaction reported in Scheme 4 required the use of 1.0 g of TsCl (5.58 mmol, 1.0 equiv), 1.0 equiv of 1‐phenylpiperazine (5.58 mmol), and 2.0 equiv (11.16 mmol) of TEA solubilized in 13 mL of solvent. This procedure led to the desired product in 87% yield (1.54 g). Reaction quenching and washing of the solid precipitate led to the generation of 52.94 g of waste, leading eventually to an E‐factor of 34. The same reaction performed in DCM, described in Section 2.2, gave instead an E‐factor of 102 (1.66 g of product **9**, 169 g of wastes for reaction quenching and extraction). The environmental factor obtained in Cyrene for this preliminary example is proving the potential of Cyrene with respect to classic organic solvents, especially considering that processes aiming at the production of fine chemicals can have E‐factors exceeding 50, while in the pharmaceutical industry, E‐factors can surpass 400. This preliminary example further proves. Given the position of this reaction within the chemical industry, we can conclude that a meaningful improvement in environmental impact has been achieved [22].

### 2.4 Cyrene Recovery

**FIGURE 3:**
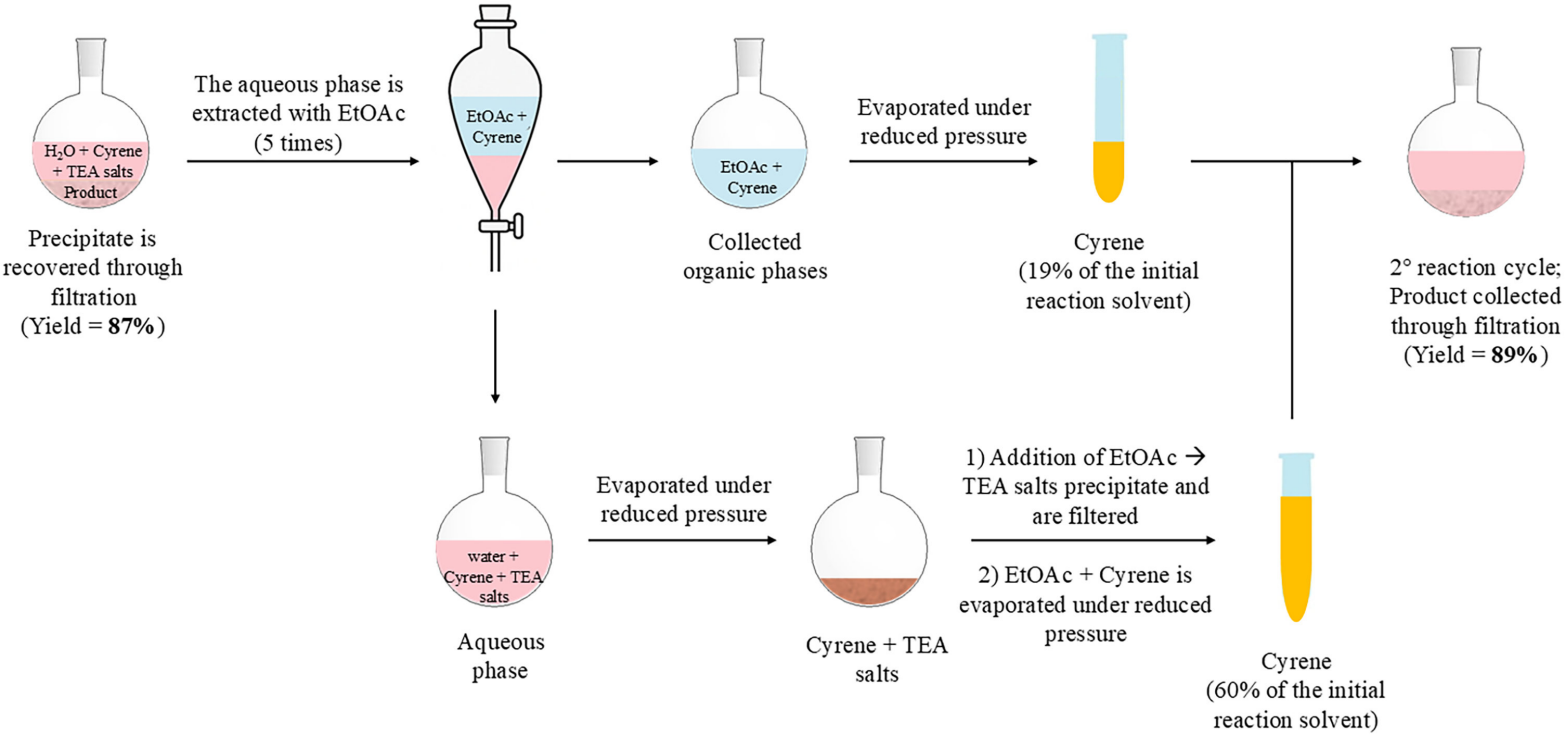
Schematic representation of the procedure followed to recover Cyrene after the product precipitation. The second cycle of the reaction proceeded without loss of yield (89% versus 87%).

**FIGURE 4:**
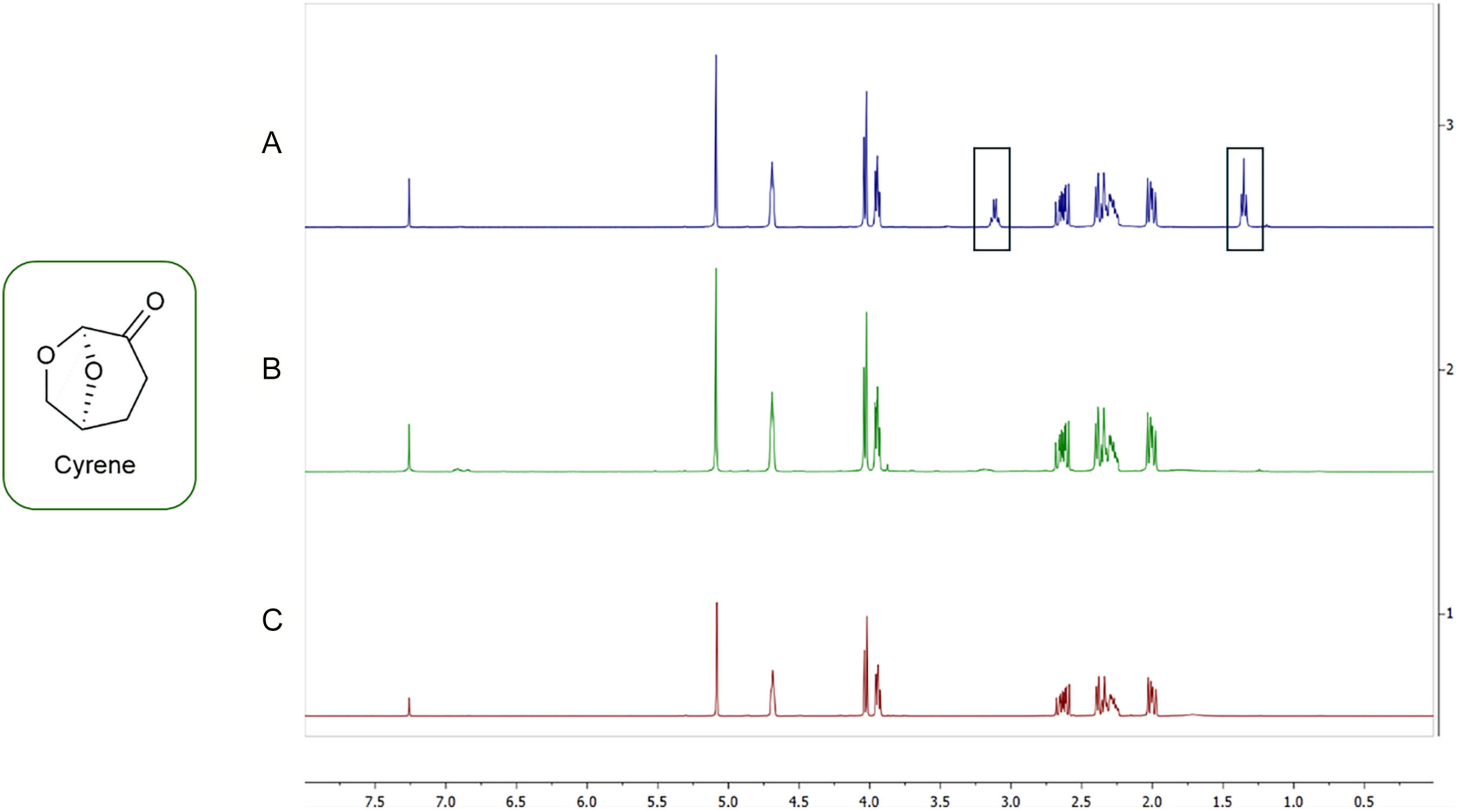
^1^H NMR spectra (CDCl_3_, 400 MHz): (A) Cyrene recovered from the aqueous phase (the signals of the TEA salts are rounded); (B) combined Cyrene recovered from the organic phase and from the aqueous phase after filtration; and (C) commercial Cyrene used as control.

To further improve the sustainability of the protocol, the recovery of Cyrene after the product precipitation was investigated (Figure 3). The reaction of TsCl with 1‐phenyl piperazine was performed in Cyrene (10 mL), as already reported in Scheme 4. After the precipitation of the sulfonamide product, the filtration/washing water was extracted five times with EtOAc: The combined organic phases were dried over anhydrous Na_2_SO_4_ and evaporated under reduced pressure, resulting in partial Cyrene recovery (19%, 2.47 mL). The use of ethyl acetate in the workup procedure is consistent with the development of a sustainable sulfonamide synthesis, as EtOAc is regarded as an extraction solvent with favorable and promising sustainability characteristics [23, 24]. Considering the high miscibility of Cyrene with water [9], in order to improve the efficiency of the recovery protocol, the aqueous phase containing the remaining Cyrene was concentrated under reduced pressure. NMR analysis of the residue showed the presence of TEA salts, as expected, due to the addition of aqueous NaHSO_4_ solution to allow the product precipitation (Figure 4A). The suspension was therefore solubilized in EtOAc and filtered to remove the solid. In this way, a further 7.80 mL of the Cyrene reaction solvent could be recovered (overall 79% recovery yield). ^1^H NMR analysis (Figure 4B) shows that the recovered solvent exhibits spectral signals identical to those of commercial Cyrene (Figure 4C), proving that the solvent was recovered pure and chemically unaltered. A second cycle of the same reaction was then performed using the recovered Cyrene, leading to the sulfonamide product with a yield almost identical to that of the first cycle (first cycle, 87%; second cycle, 89%), meaning that the recovery process does not affect the efficiency of Cyrene as reaction solvent (Figure 2).

## 3 Conclusions

The experimental protocol developed for the synthesis of sulfonamides in Cyrene proved to be efficient, simple, and rapid. All products were isolated through precipitation, without the need for chromatographic purification. The use of Cyrene allowed the reactions to reach completion within shorter reaction times (30–60 min) affording the desired products in moderate to high yields. Compared to procedures reported in the literature, the present protocol exhibits broad applicability, as demonstrated by the wide range of amines evaluated, while consistently operating under exceptionally mild conditions. Reaction scalability was further validated through a model reaction performed on a 1.0 g scale. An efficient solvent recovery procedure was also established, and ^1^H NMR analysis confirmed that Cyrene could be recycled without any alterations of its chemical structure. Notably, the recycled solvent was successfully reused in a subsequent reaction cycle, providing the product in yields comparable to those obtained in the first cycle. An assessment of the environmental impact, based on the E‐factor, revealed that Cyrene‐based protocol reduces waste generation by nearly fivefold compared to the conventional DCM‐based method. Overall, this protocol represents a versatile and efficient approach for sulfonamide synthesis, combining broad substrate scope with a significantly improved environmental profile.

## 4 Experimental Section

### 4.1 Materials and Methods

Unless otherwise stated, reagents and solvents were purchased from Merck (Milan, Italy), Fluorochem (Hadfield, United Kingdom), or BLDPharm (Reinbek, Germany) and used without further purification. All reactions were carried out in an oven‐dried glass‐ware, using dry solvents, and monitored by TLC on silica gel (Merck precoated 60 F254 plates), with detection by UV light (254 nm). Purity of the final compounds was assured to be >95% as assessed by NMR. ^1^H NMR and ^13^C NMR spectra were recorded at 298 K on a Brüker Avance Spectrometer (400 MHz), using commercially available deuterated solvents (DMSO‐*d*_6_, Chloroform‐*d*). Chemical shifts are reported in parts per million (*δ* ppm), compared to TMS as an internal standard. Coupling constants (*J*) are given in hertz (Hz) and are quoted to the nearest 0.5 Hz. Peak multiplicities are described in the following way: s, singlet; bs, broad singlet; d, doublet; m, multiplet; and br, broad. Melting points of the final compounds were determined using a “Stuart SMP3 Apparatus.”

### 4.2 General Synthetic Procedure

The appropriate sulfonyl chloride (1.0 mmol, 0.25 M), amine (1.0 mmol) and base (refer Notes below) were dissolved in Cyrene, and the reaction was left stirring at room temperature until reaction completion as observed by TLC (30 min–1 h). The reaction mixture was cooled to room temperature, and a mixture of water, ice, and aqueous HCl 1 M or NaHSO_4_ 0.5 M was added. The resulting precipitate was collected by filtration and washed with water to afford the pure product.

*Notes*:

Products **1**–**3**: Base = NaHCO_3_ (1.5 mmol); the reaction was quenched with HCl 1.0 M.
Products **4**–**8**: Base = pyridine (2.1 mmol); the reaction was quenched with HCl 1.0 M.
Products **9**, **13**–**20**: Base = TEA (2.0 mmol); the reaction was quenched with NaHSO_4_ 0.5 M.
Products **10**–**12**: Base = pyridine (2.0 mmol); the reaction was quenched with NaHSO_4_ 0.5 M.

#### 4.2.1 *N*‐Benzyl‐4‐Methylbenzenesulfonamide (1)

By following the general procedure, starting from *p*‐toluenesulfonyl chloride (190 mg, 1.0 mmol, 1.0 equiv), benzylamine (107 mg, 1.0 mmol, 1.0 equiv), and NaHCO_3_ (126 mg, 1.5 mmol, 1.5 equiv) in Cyrene (4 mL). Compound **1** was obtained with a 79% yield as a white solid; m.p. 116–118 °C (lit.: 115–118 °C). ^1^H NMR (400 MHz, Chloroform‐*d*): *δ* 7.79 (d, *J* = 8.4 Hz, 2H), 7.37–7.24 (m, 5H), 7.25–7.19 (m, 2H), 4.72 (s, 1H), 4.15 (d, *J* = 6.2 Hz, 2H), 2.47 (s, 3H). Spectral and analytical characterizations are in agreement with the ones reported in the literature [25].

#### 4.2.2 *N*‐(4‐Methoxybenzyl)‐4‐Methylbenzenesulfonamide (2)

By following the general procedure, starting from *p*‐toluenesulfonyl chloride (190 mg, 1.0 mmol, 1.0 equiv), 4‐methoxyaniline (137 mg, 1.0 mmol, 1.0 equiv), and NaHCO_3_ (126 mg, 1.5 mmol, 1.5 equiv) in Cyrene (4 mL). Compound **2** was obtained with a 81% yield as a light brown solid; m.p. 118–119 °C (lit.: 118–119 °C). ^1^H NMR (400 MHz, Chloroform‐*d*) δ 7.75 (d, *J* = 8.4 Hz, 2H), 7.31 (d, *J* = 8.6 Hz, 2H), 7.10 (d, *J* = 8.8 Hz, 2H), 6.79 (d, *J* = 8.8 Hz, 2H), 4.05 (d, *J* = 6.1 Hz, 2H), 3.77 (s, 3H), 2.44 (s, 3H). Spectral and analytical characterizations are in agreement with the ones reported in the literature [26].

#### 4.2.3 4‐Methyl‐*N*‐(2‐Pyridinylmethyl)‐Benzenesulfonamide (3)

By following the general procedure, starting from *p*‐toluenesulfonyl chloride (190 mg, 1.0 mmol, 1.0 equiv), 2‐picolylamine (108 mg, 1.0 mmol, 1.0 equiv), and triethylamine (0.28 mL, 2.0 mmol, 2.0 equiv) in Cyrene (4 mL). Compound **3** was obtained with a 31% yield as a light brown solid; m.p. 88–91 °C (lit.: 92–94 °C). ^1^H NMR (400 MHz, DMSO‐*d*_6_) *δ* 8.43 (d, *J* = 4.0 Hz, 1H), 8.17 (t, *J* = 6.3 Hz, 1H), 7.73 (td, *J* = 7.6, 1.8 Hz, 1H), 7.68 (d, *J* = 8.3 Hz, 2H), 7.37 (d, *J* = 7.9 Hz, 2H), 7.34 (s, 1H), 7.24 (dd, *J* = 7.6, 4.8 Hz, 1H), 4.04 (d, *J* = 6.5 Hz, 2H), 2.38 (s, 3H). Spectral and analytical characterizations are in agreement with the ones reported in the literature [27].

#### 4.2.4 *N*‐(4‐Bromophenyl)‐4‐Methylbenzenesulfonamide (4)

By following the general procedure, starting from *p*‐toluenesulfonyl chloride (190 mg, 1.0 mmol, 1.0 equiv), 4‐bromoaniline (172 mg, 1.0 mmol, 1.0  equiv) and pyridine (0.17 mL, 2.1 mmol, 2.1  equiv) in Cyrene (4 mL). Compound **4** was obtained with a 64% yield as a light brown solid; m.p. 138–140 °C (lit: 142–143 °C). ^1^H NMR (400 MHz, Chloroform‐*d*) *δ* 7.62 (d, *J* = 8.3 Hz, 2H), 7.36 (d, *J* = 8.9 Hz, 2H), 7.24 (d, *J* = 8.6 Hz, 2H), 6.94 (d, *J* = 8.8 Hz, 2H), 6.38 (s, 1H), 2.39 (s, 3H). Spectral and analytical characterizations are in agreement with the ones reported in the literature [28].

#### 4.2.5 *N*‐(4‐Methoxyphenyl)‐4‐Methylbenzenesulfonamide (5)

By following the general procedure, starting from *p*‐toluenesulfonyl chloride (190 mg, 1.0 mmol, 1.0 equiv), 4‐methoxyaniline (123 mg, 1.0 mmol, 1.0 equiv), and pyridine (0.17 mL, 2.1 mmol, 2.1 equiv) in Cyrene (4 mL). Compound **5** was obtained with a 50% yield as a light brown solid; m.p. 98–100 °C (lit: 101–103 °C). ^1^H NMR (400 MHz, Chloroform‐*d*) *δ* 7.58 (d, *J* = 7.9 Hz, 2H), 7.21 (d, *J* = 7.9 Hz, 2H), 6.97 (d, *J* = 8.4 Hz, 2H), 6.76 (d, *J* = 8.4 Hz, 2H), 6.45 (s, 1H), 3.75 (s, 3H), 2.38 (s, 3H). Spectral and analytical characterizations are in agreement with the ones reported in the literature [29].

#### 4.2.6 *N*‐(4‐Acetylphenyl)‐4‐Methylbenzenesulfonamide (6)

By following the general procedure, starting from *p*‐toluenesulfonyl chloride (190 mg, 1.0 mmol, 1.0 equiv), 4‐aminoacetophenone (135 mg, 1.0 mmol, 1.0 equiv), and pyridine (0.17 mL, 2.1 mmol, 2.1 equiv) in Cyrene (4 mL). Compound **6** was obtained with a 63% yield as a light brown solid; m.p. 202–204 °C (lit.: 203–205 °C). ^1^H NMR (400 MHz, Chloroform‐*d*) *δ* 7.84 (d, *J* = 8.8 Hz, 2H), 7.73 (d, *J* = 8.4 Hz, 2H), 7.25 (d, *J* = 5.8 Hz, 2H), 7.15 (d, *J* = 8.8 Hz, 2H), 7.10 (s, 1H), 2.53 (s, 3H), 2.38 (s, 3H). Spectral and analytical characterizations are in agreement with the ones reported in the literature [30].

#### 4.2.7 4‐(Toluene‐4‐Sulfonylamino)‐Benzoic Acid Methyl Ester (7)

By following the general procedure, starting from *p*‐toluenesulfonyl chloride (190 mg, 1.0 mmol, 1.0 equiv), methyl 4‐aminobenzoate (151 mg, 1.0 mmol, 1.0 equiv), and pyridine (0.17 mL, 2.1 mmol, 2.1 equiv) in Cyrene (4 mL). Compound **7** was obtained with a 65% yield as a light brown solid; m.p. 195–197 °C (lit.: 193–195 °C). ^1^H NMR (400 MHz, Chloroform‐*d*) *δ* 7.91 (d, *J* = 8.8 Hz, 2H), 7.72 (d, *J* = 8.4 Hz, 2H), 7.24 (d, *J* = 7.9 Hz, 2H), 7.18 (s, 1H), 7.13 (d, *J* = 8.8 Hz, 2H), 3.87 (s, 3H), 2.37 (s, 3H). Spectral and analytical characterizations are in agreement with the ones reported in the literature [31].

#### 4.2.8 *N*‐((4‐(Acetylamino)phenyl)sulphonyl)acetamide (8)

By following the general procedure, starting from 4‐acetamidobenzenesulfonyl chloride (234 mg, 1.0 mmol, 1.0 equiv), 4′‐aminoacetophenone (135 mg, 1.0 mmol, 1.0 equiv), and pyridine (0.17 mL, 2.1 mmol, 2.1 equiv) in Cyrene (4 mL). Compound **8** was obtained with a 17% yield as a light brown solid; m.p. 234–236 °C (lit.: 238–240 °C). ^1^H NMR (400 MHz, DMSO‐*d*_6_): *δ* 10.74 (s, 1H), 10.30 (s, 1H), 7.83 (d, *J* = 8.3 Hz, 2H), 7.75 (q, *J* = 8.6 Hz, 4H), 7.20 (d, *J* = 8.5 Hz, 2H), 2.46 (s, 3H), 2.05 (s, 3H). ^13^C NMR (100 MHz, DMSO‐*d*_6_): *δ* 196.2, 168.8, 143.2, 142.1, 132.4, 131.6, 129.5, 127.8, 118.5, 117.6, 26.1, 23.9. HRMS (ESI): *m/z* [M + Na]^+^ calculated for C_16_H_16_N_2_O_4_SNa^+^: 355.0723; found: 355.0774.

#### 4.2.9 1‐Phenyl‐4‐Tosylpiperazine (9)

By following the general procedure, starting from *p*‐toluenesulfonyl chloride (190 mg, 1.0 mmol, 1.0 equiv), 1‐phenylpiperazine (162 mg, 1.0 mmol, 1.0 equiv), and triethylamine (0.28 mL, 2.0 mmol, 2.0 equiv) in Cyrene (4 mL). Compound **9** was obtained with a 90% yield as a light brown solid; m.p. 195–196 °C (lit.: 198–200 °C). ^1^H NMR (400 MHz, Chloroform‐*d*) *δ* 7.68 (d, *J* = 8.3 Hz, 2H), 7.34 (d, *J* = 8.6 Hz, 2H), 7.24 (d, *J* = 8.8 Hz, 2H), 6.90 (d, *J* = 7.2 Hz, 1H), 6.88–6.85 (m, 2H), 3.26–3.22 (m, 4H), 3.16 (t, *J* = 4.9 Hz, 4H), 2.44 (s, 3H). Spectral and analytical characterizations are in agreement with the ones reported in the literature [32].

#### 4.2.10 2‐(4‐Tosylpiperazin‐1‐yl)phenol (10)

By following the general procedure, starting from *p*‐toluenesulfonyl chloride (190 mg, 1.0 mmol, 1.0 equiv), 2‐(piperazin‐1‐yl)phenol (178 mg, 1.0 mmol, 1.0 equiv), and pyridine (0.17 mL, 2.0 mmol, 2.0 equiv) in Cyrene (4 mL). Compound **10** was obtained with a 50% yield as a light brown solid; m.p. 180–182 °C. ^1^H NMR (400 MHz, Chloroform‐*d*) *δ* 7.69 (d, *J* = 8.3 Hz, 2H), 7.38 (d, *J* = 8.1 Hz, 2H), 7.14–7.05 (m, 2H), 3.18 (s, 4H), 2.96 (t, *J* = 4.9 Hz, 4H), 2.47 (s, 3H).^13^C NMR (100 MHz, Chloroform‐*d*) *δ* 151.3, 144.1, 138.1, 132.6, 130.0, 129.9, 128.0, 127.1, 121.6, 121.5, 120.4, 114.5, 51.9, 46.9, 46.9, 21.7. HRMS (ESI): *m/z* [M + H]^+^ calculated for C_17_H_21_N_2_O_3_S^+^: 333.1267; found: 333.1191.

#### 4.2.11 3‐(4‐Tosylpiperazin‐1‐yl)phenol (11)

By following the general procedure, starting from *p*‐toluenesulfonyl chloride (190 mg, 1.0 mmol, 1.0 equiv), 3‐(piperazin‐1‐yl)phenol (178 mg, 1.0 mmol, 1.0 equiv), and pyridine (0.17 mL, 2.0 mmol, 2.0 equiv) in Cyrene (4 mL). Compound **11** was obtained with a 51% yield as a light brown solid; m.p. 175–177 °C. ^1^H NMR (400 MHz, DMSO‐*d*_6_) *δ* 7.64 (d, *J* = 7.8 Hz, 2H), 7.47 (d, *J* = 8.0 Hz, 2H), 6.96 (t, *J* = 8.0 Hz, 1H), 6.32 (d, *J* = 8.2 Hz, 1H), 6.29–6.15 (m, 2H), 3.20–3.09 (m, 4H), 2.95 (t, *J* = 4.9 Hz, 4H), 2.41 (s, 3H). ^13^C NMR (100 MHz, Chloroform‐*d*) *δ* 151.3, 144.1, 138.1, 132.6, 130.0, 129.9, 128.0, 127.1, 121.6, 121.5, 120.4, 114.5, 51.9, 46.9, 46.9, 21.7. HRMS (ESI): *m/z* [M + H]^+^ calculated for C_17_H_21_N_2_O_3_S^+^: 333.1267; found: 333.1203.

#### 4.2.12 4‐(4‐Tosylpiperazin‐1‐yl)phenol (12)

By following the general procedure, starting from *p*‐toluenesulfonyl chloride (190 mg, 1.0 mmol, 1.0 equiv), 4‐(piperazin‐1‐yl)phenol (178 mg, 1.0 mmol, 1.0 equiv), and pyridine (0.17 mL, 2.0 mmol, 2.0 equiv) in Cyrene (4 mL). Compound **12** was obtained with a 42% yield as a light brown solid; m.p. 179–181 °C. ^1^H NMR (400 MHz, Chloroform‐*d*) *δ* 7.67 (d, *J* = 8.2 Hz, 2H), 7.34 (d, *J* = 8.0 Hz, 2H), 6.76 (q, *J* = 9.1 Hz, 4H), 3.18–3.06 (m, 8H), 2.44 (s, 3H). ^13^C NMR (100 MHz, Chloroform‐*d*): *δ* 150.9, 144.8, 144.0, 132.4, 129.9, 128.0, 119.5, 116.1, 50.8, 46.3, 21.6. HRMS (ESI): *m/z* [M + H]^+^ calculated for C_17_H_21_N_2_O_3_S^+^: 333.1267; found: 333.1140.

#### 4.2.13 1‐(3‐Methoxyphenyl)‐4‐Tosylpiperazine (13)

By following the general procedure, starting from *p*‐toluenesulfonyl chloride (190 mg, 1.0 mmol, 1.0 equiv), 1‐(3‐methoxyphenyl)piperazine (192 mg, 1.0 mmol, 1.0 equiv), and triethylamine (0.28 mL, 2.0 mmol, 2.0 equiv) in Cyrene (4 mL). Compound **13** was obtained with a 67% yield as a light brown solid; m.p. 154–156 °C. ^1^H NMR (400 MHz, Chloroform‐*d*): *δ* 7.69 (d, *J* = 8.4 Hz, 2H), 7.37 (d, *J* = 8.1 Hz, 2H), 7.25 (t, *J* = 8.4 Hz, 1H), 6.73–6.60 (m, 3H), 3.81 (s, 3H), 3.37 (s, 8H), 2.46 (s, 3H). ^13^C NMR (100 MHz, Chloroform‐*d*): *δ* 160.7, 144.2, 132.5, 130.4, 129.9, 127.8, 110.5, 104.6, 96.7, 55.4, 50.5, 45.10, 21.6. HRMS (ESI): *m/z* [M + H]^+^ calculated for C_18_H_23_N_2_O_3_S^+^: 347.1424; found: 347.1486.

#### 4.2.14 1‐(4‐Methoxyphenyl)‐4‐Tosylpiperazine (14)

By following the general procedure, starting from *p*‐toluenesulfonyl chloride (190 mg, 1.0 mmol, 1.0 equiv), 1‐(4‐methoxyphenyl)piperazine (192 mg, 1.0 mmol, 1.0 equiv), and triethylamine (0.28 mL, 2.0 mmol, 2.0 equiv) in Cyrene (4 mL). Compound **14** was obtained with a 32% yield as a light brown solid; m.p. 167–169 °C. ^1^H NMR (400 MHz, Chloroform‐*d*): *δ* 7.67 (d, *J* = 8.3 Hz, 2H), 7.34 (d, *J* = 8.6 Hz, 2H), 6.83 (d, *J* = 2.8 Hz, 4H), 3.75 (s, 3H), 3.14 (d, *J* = 6.8 Hz, 8H), 2.44 (s, 3H).^13^C NMR (100 MHz, Chloroform‐*d*): *δ* 156.3, 144.0, 132.5, 129.9, 128.0, 119.4, 114.7, 55.7, 51.2, 46.6, 21.7. HRMS (ESI): *m/z* [M + H]^+^ calculated for C_18_H_23_N_2_O_3_S^+^: 347.1424; found: 347.1455.

#### 4.2.15 1‐Phenyl‐4‐Tosylpiperazine (15)

By following the general procedure, starting from *p*‐toluenesulfonyl chloride (190 mg, 1.0 mmol, 1.0 equiv), 1‐(2,6‐dimethylphenyl)piperazine hydrochloride (227 mg, 1.0 mmol, 1.0 equiv), and triethylamine (0.28 mL, 2.0 mmol, 2.0 equiv) in Cyrene (4 mL). Compound **15** was obtained with a 20% yield as a light brown solid; m.p. 175–177 °C (lit.: 172–173 °C). ^1^H NMR (400 MHz, Chloroform‐*d*): *δ* 7.69 (d, *J* = 8.0 Hz, 2H), 7.36 (d, *J* = 8.0 Hz, 2H), 6.96 (s, 3H), 3.19–3.13 (m, 4H), 3.14–3.07 (m, 4H), 2.47 (s, 3H), 2.23 (s, 6H). Spectral and analytical characterizations are in agreement with the ones reported in the literature [33].

#### 4.2.16 1‐(Benzo[*d* [1,3]dioxol‐5‐Ylmethyl)‐4‐Tosylpiperazine (16)

By following the general procedure, starting from *p*‐toluenesulfonyl chloride (190 mg, 1.0 mmol, 1.0 equiv), 1‐(benzo[*d* [1,3]dioxol‐5‐ylmethyl)piperazine (220 mg, 1.0 mmol, 1.0 equiv), and triethylamine (0.28 mL, 2.0 mmol, 2.0 equiv) in Cyrene (4 mL). Compound **16** was obtained with a 57% yield as a light brown solid; m.p. 144–146 °C (lit.: 146–147 °C). ^1^H NMR (400 MHz, CDCl3) *δ* 7.62 (d, *J* = 8.3 Hz, 2H), 7.32 (d, *J* = 8.0 Hz, 2H), 6.75 (s, 1H), 6.69 (q, *J* = 7.8 Hz, 2H), 5.91 (s, 2H), 3.40 (s, 2H), 3.01 (s, 4H), 2.51 (s, 4H), 2.43 (s, 3H). Spectral and analytical characterizations are in agreement with the ones reported in the literature [34].

#### 4.2.17 1‐(4‐Fluorobenzyl)‐4‐Tosylpiperazine (17)

By following the general procedure, starting from *p*‐toluenesulfonyl chloride (190 mg, 1.0 mmol, 1.0 equiv), 1‐(4‐fluorobenzyl)piperazine (194 mg, 1.0 mmol, 1.0 equiv), and triethylamine (0.28 mL, 2.0 mmol, 2.0 equiv) in Cyrene (4 mL). Compound **17** was obtained with a 39% yield as a light brown solid; m.p. 156–158 °C ^1^H NMR (400 MHz, CDCl_3_) *δ* 7.62 (d, *J* = 8.1 Hz, 2H), 7.32 (d, *J* = 8.1 Hz, 2H), 7.21–7.16 (m, 2H), 6.96 (t, *J* = 8.7 Hz, 2H), 3.44 (s, 2H), 3.01 (s, 4H), 2.50 (s, 4H), 2.43 (s, 3H). Spectral and analytical characterizations are in agreement with the ones reported in the literature [35].

#### 4.2.18 1‐Methyl‐4‐Tosylpiperazine (18)

By following the general procedure, starting from *p*‐toluenesulfonyl chloride (190 mg, 1.0 mmol, 1.0 equiv), 1‐methylpiperazine (100 mg, 1.0 mmol, 1.0 equiv) and triethylamine (0.28 mL, 2.0 mmol, 2.0 equiv) in Cyrene (4 mL). Compound **18** was obtained with a 17% yield as a light brown solid; m.p. 144–146 °C (lit.: 140–142 °C) ^1^H NMR (400 MHz, Chloroform‐*d*) *δ* 7.63 (d, *J* = 8.3 Hz, 2H), 7.31 (d, *J* = 8.0 Hz, 2H), 3.01 (s, 4H), 2.47 (t, *J* = 5.0 Hz, 4H), 2.42 (s, 3H), 2.26 (s, 3H). Spectral and analytical characterizations are in agreement with the ones reported in the literature [36].

#### 4.2.19 *N*’‐Tosyl‐*N*‐Formyl‐Piperazine (19)

By following the general procedure, starting from *p*‐toluenesulfonyl chloride (190 mg, 1.0 mmol, 1.0 equiv), piperazine‐1‐carbaldehyde (114 mg, 1.0 mmol, 1.0 equiv), and triethylamine (0.28 mL, 2.0 mmol, 2.0 equiv) in Cyrene (4 mL). Compound **19** was obtained with a 12% yield as a light brown solid; m.p. 135–137 °C. ^1^H NMR (400 MHz, Chloroform‐*d*) *δ* 7.95 (s, 1H), 7.59 (t, *J* = 9.4 Hz, 2H), 7.32 (d, *J* = 7.8 Hz, 2H), 3.62 (s, 2H), 3.45 (s, 2H), 3.08–2.97 (m, 4H), 2.42 (s, 3H). ^13^C NMR (100 MHz, Chloroform‐*d*) *δ* 160.7, 144.4, 132.5, 130.0, 127.8, 46.7, 45.6, 45.1, 39.4, 21.6. HRMS (ESI): *m/z* [M + Na]^+^ calculated for C_12_H_16_N_2_O_3_SNa^+^: 291.0774; found: 291.0685.

#### 4.2.20 1‐Boc‐4‐Tosylpiperazine (20)

By following the general procedure, starting from *p*‐toluenesulfonyl chloride (190 mg, 1.0 mmol, 1.0 equiv), 1‐Boc‐piperazine (186 mg, 1.0 mmol, 1.0 equiv), and triethylamine (0.28 mL, 2.0 mmol, 2.0 equiv) in Cyrene (4 mL). Compound **20** was obtained with a 25% yield as a light brown solid; m.p. 155–157 °C (lit.: 158–160 °C). ^1^H NMR (400 MHz, Chloroform‐*d*) *δ* 7.63 (d, *J* = 8.2 Hz, 2H), 7.33 (d, *J* = 8.3 Hz, 2H), 3.50 (s, 4H), 2.95 (s, 4H), 2.43 (s, 3H), 1.40 (s, 9H). Spectral and analytical characterizations are in agreement with the ones reported in the literature [36].

#### 4.2.21 Cyrene

Recovered from the reaction mixture as described in paragraph 2.6. ^1^H NMR (400 MHz, Chloroform‐*d*) *δ* 5.09 (s, 1H), 4.69 (dt, *J* = 5.1, 2.4 Hz, 1H), 4.03 (dd, *J* = 7.4, 1.1 Hz, 1H), 3.95 (ddd, *J* = 7.1, 5.1, 1.6 Hz, 1H), 2.64 (ddd, *J* = 16.1, 11.6, 8.5 Hz, 1H), 2.44–2.23 (m, 2H), 2.06–1.96 (m, 1H).

### 4.3 Gram‐Scale Procedure

*p*‐Toluenesulfonyl chloride (5.58 mmol), 1‐phenylpiperazine (5.58 mmol), and triethylamine (11.16 mmol) were dissolved in Cyrene (13 mL), and the reaction was left stirring at room temperature for 30 min. The reaction mixture was cooled to 0 °C, and a mixture of water, ice, and aqueous NaHSO_4_ 0.5 M was added. The resulting precipitate was collected by filtration and washed with water to afford the pure product (1.536 g, 87% yield).

### 4.4 Cyrene Recovery Procedure

The reaction between *p*‐toluenesulfonyl chloride and 1‐phenylpiperazine was performed as described in Section 4.3. The aqueous phase obtained after the product’s filtration and washing was extracted with EtOAc (5 × 20 mL). The collected organic phases were dried over anhydrous Na_2_SO_4_ and evaporated under reduced pressure to give chemically unaltered Cyrene (1.9 mL, 19% of the reaction solvent). The aqueous phase was evaporated itself under reduced pressure, and the obtained crude was solubilized in EtOAc, in which the TEA salts generated during the reaction quenching precipitated. The solid was removed through filtration, and the organic phase was evaporated under reduced pressure to give chemically unaltered Cyrene (6.0 mL, 60% of the initial reaction solvent).

## Author Contributions

**Michela Galli:** writing review and editing, writing original draft, visualization, project administration, methodology, investigation, formal analysis, data curation, conceptualization. **Andrea Fulminante:** investigation, data curation. **Alessandro Fiori:** investigation, data curation. **Valerio Fasano:** supervision, resources, project administration, investigation. **Alessandra Silvani:** writing original draft, supervision, resources, methodology, investigation, formal analysis. **Daniele Passarella:** writing original draft, visualization, software, methodology, investigation, formal analysis, resources, funding acquisition and validation. **Andrea Citarella:** writing review and editing, writing original draft, visualization, project administration, methodology, investigation, formal analysis, data curation, conceptualization.

## Funding

This work was funded by Regione Lombardia and cofinanced by European Union ‐ Call “Collabora & Innova” – Project 6154644 GREEN‐TECH (“Applicazione di tecnologie innovative a basso impatto ambientale per lo sviluppo e l’ottimizzazione di processi chimici sostenibili”).

## Conflicts of Interest

The authors declare no conflicts of interest.

## Supporting information

The Supporting Information includes copies of ^1^H and ^13^C NMR of final compounds.

## Data Availability

The data that support the findings of this study are available from the corresponding author upon reasonable request.
