# Supplementary material for: Synthesis of Sulfonamides in the Biobased Solvent Cyrene
Source: ChemistryOpen. 2026 Aug 27;15(9):e70290. doi: 10.1002/open.70290 (PMC13519667; doi:10.1002/open.70290)
Supplement: Supplementary file 1 [file OPEN-15-e70290-s001.pdf]

Supplementary materials for:

Synthesis of sulfonamides in the bio-based solvent Cyrene^{TM}

Michela Galli,[a] Andrea Fulminante,[a] Alessandro Fiori,[a] Valerio Fasano,[a] Alessandra Silvani, [a] Daniele Passarella* [a] and Andrea Citarella* [a1]

[a] *Department of Chemistry, University of Milan, Via Golgi 19 20133 Milano, Italy*

corresponding: andrea.citarella@unica.it and daniele.passarella@unimi.it

[1] Present address*: Department of Chemical and Geological Sciences, University of Cagliari, Cittadella Universitaria di Monserrato, SS 554, bivio per Sestu, 09042 Monserrato (CA), Italy*

Contents	Pages
Materials and methods	S1
^{1}H and ^{13}C NMR Spectra	S2 – S15

1. Materials and Methods

Unless otherwise stated, reagents and solvents were purchased from Merck (Milan, Italy), Fluorochem (Hadfield, United Kingdom) or BLDPharm (Reinbek, Germany) and used without further purification. All reactions were carried out in oven-dried glass-ware, using dry solvents, and monitored by TLC on silica gel (Merck precoated 60 F254 plates), with detection by UV light (254 nm). Purity of the final compounds was assured to be >95% as assessed by NMR. ^{1}H NMR and ^{13}C NMR spectra were recorded at 298 K on a Brüker Avance Spectrometer (400 MHz), using commercially available deuterated solvents (DMSO-d_6, Chloroform-*d*). Chemical shifts are reported in parts per million (δppm), compared to TMS as an internal standard. Coupling constants (J) are given in hertz (Hz) and are quoted to the nearest 0.5 Hz. Peak multiplicities are described in the following way: s, singlet; bs, broad singlet; d, doublet; m, multiplet; br, broad. Melting points of the final compounds were determined using a "Stuart SMP3 Apparatus".

2. ^{1}H and ^{13}C NMR spectra of the final compounds

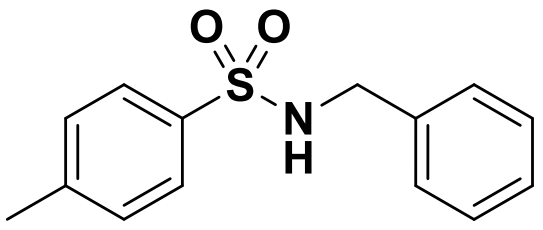

1

^{1}H NMR, 400 MHz, $CDCl_3$

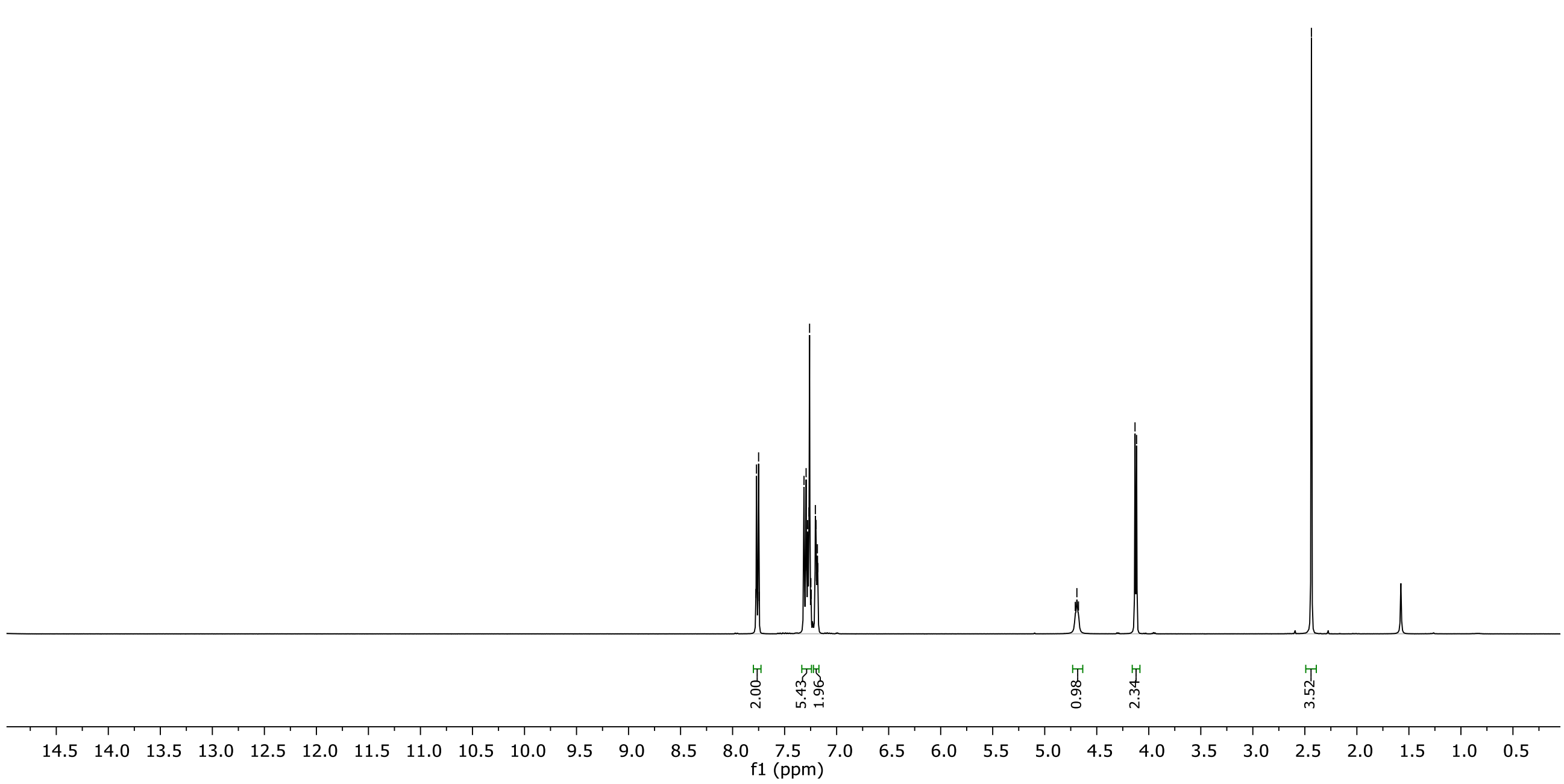

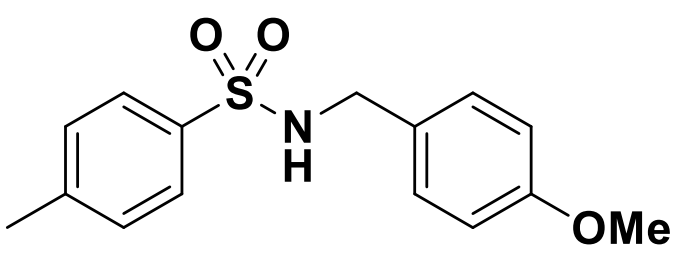

2

^{1}H NMR, 400 MHz, $CDCl_3$

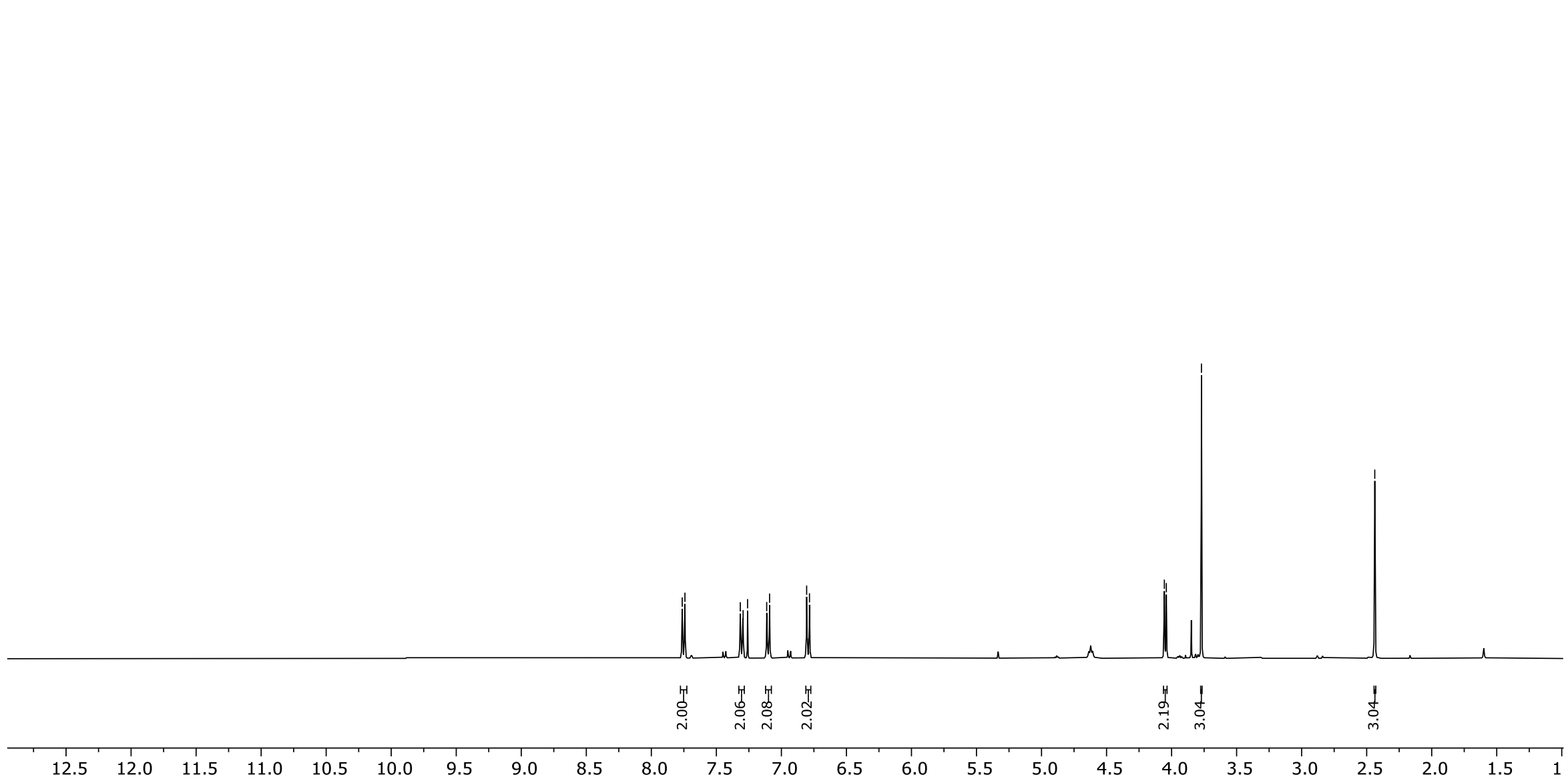

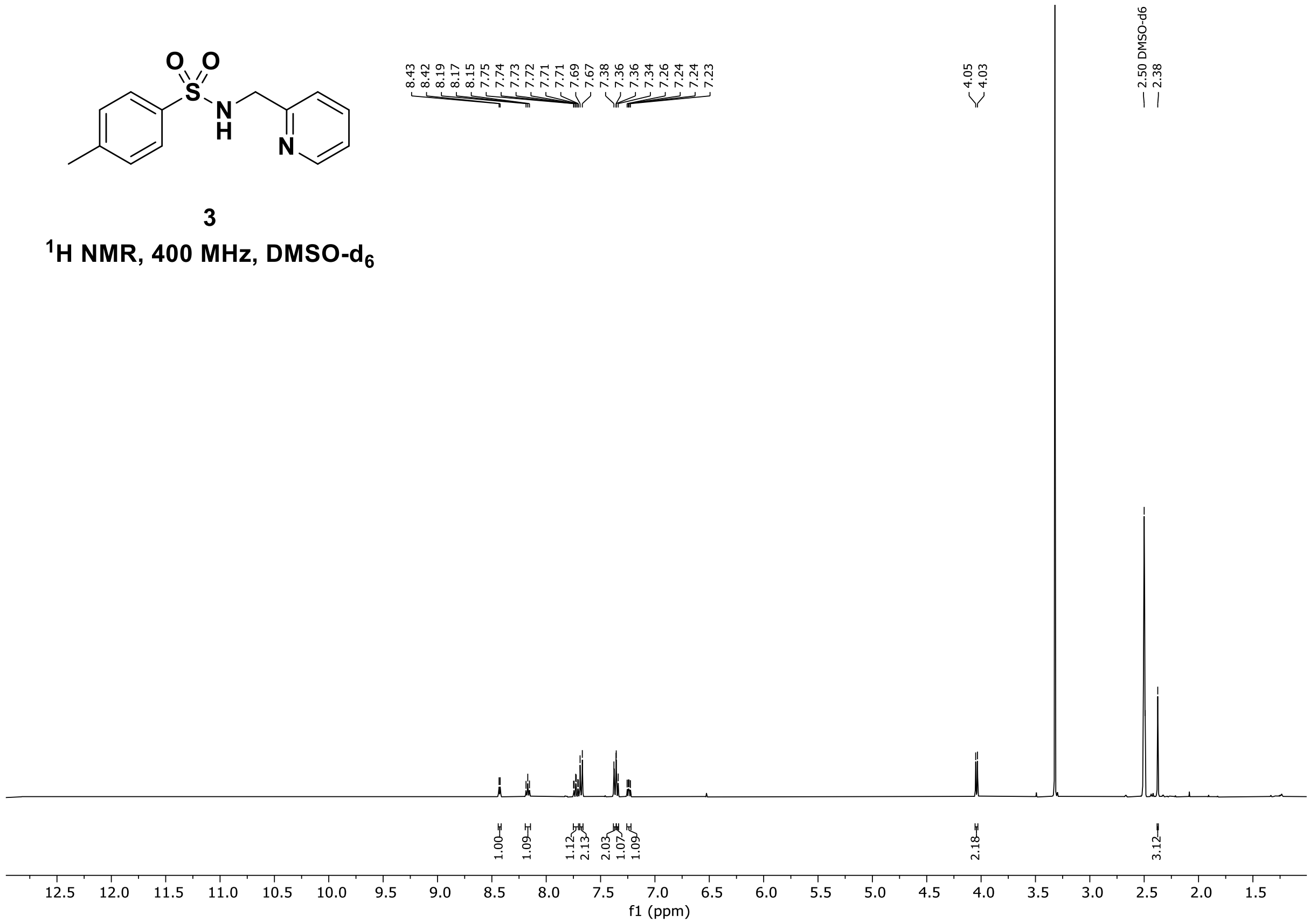

3

^{1}H NMR, 400 MHz, DMSO-d_6

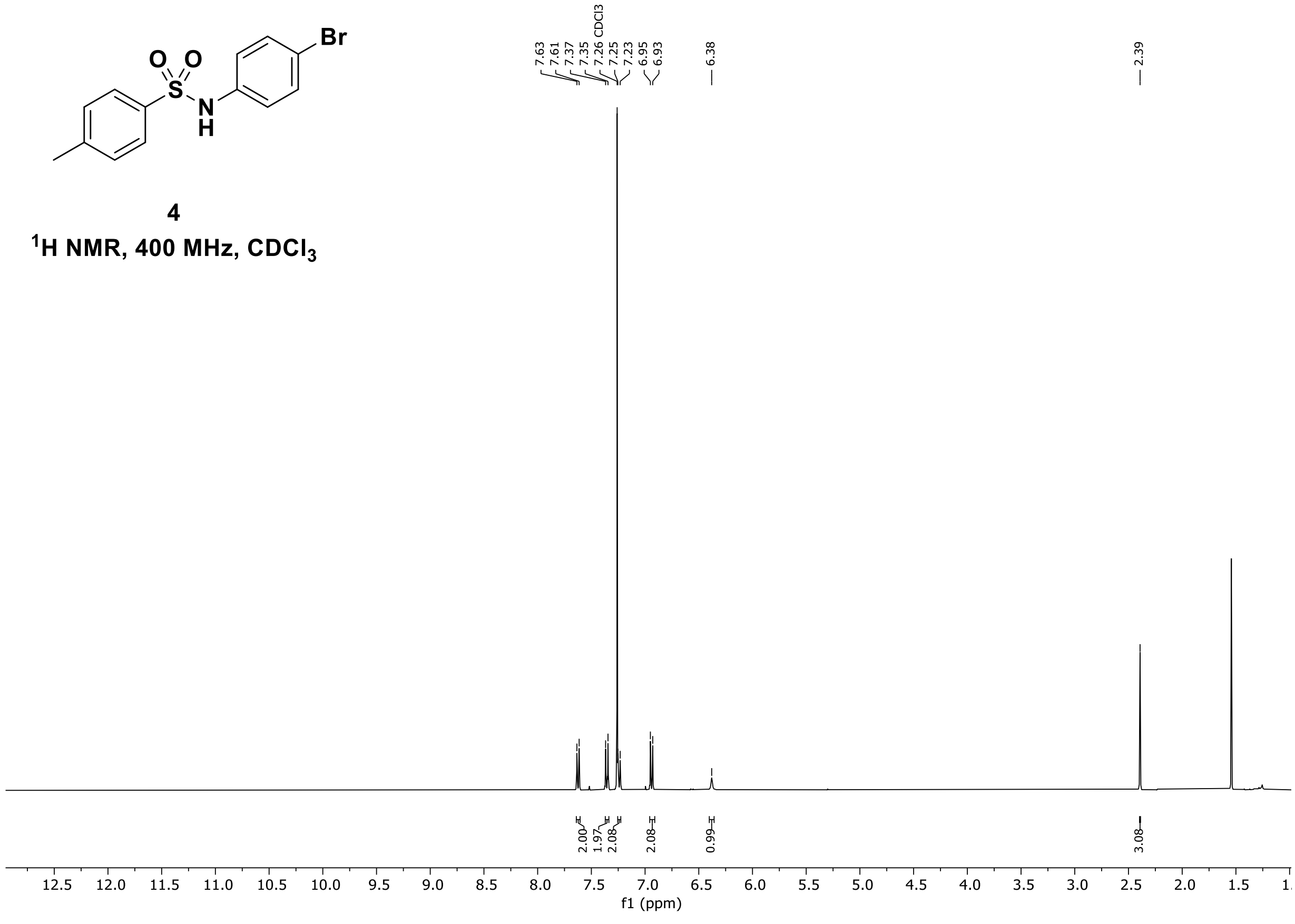

4

^{1}H NMR, 400 MHz, $CDCl_3$

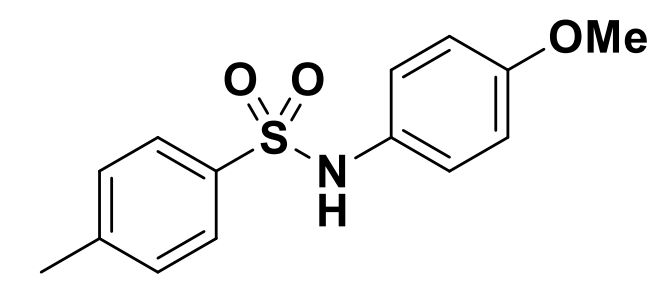

5

^{1}H NMR, 400 MHz, $CDCl_3$

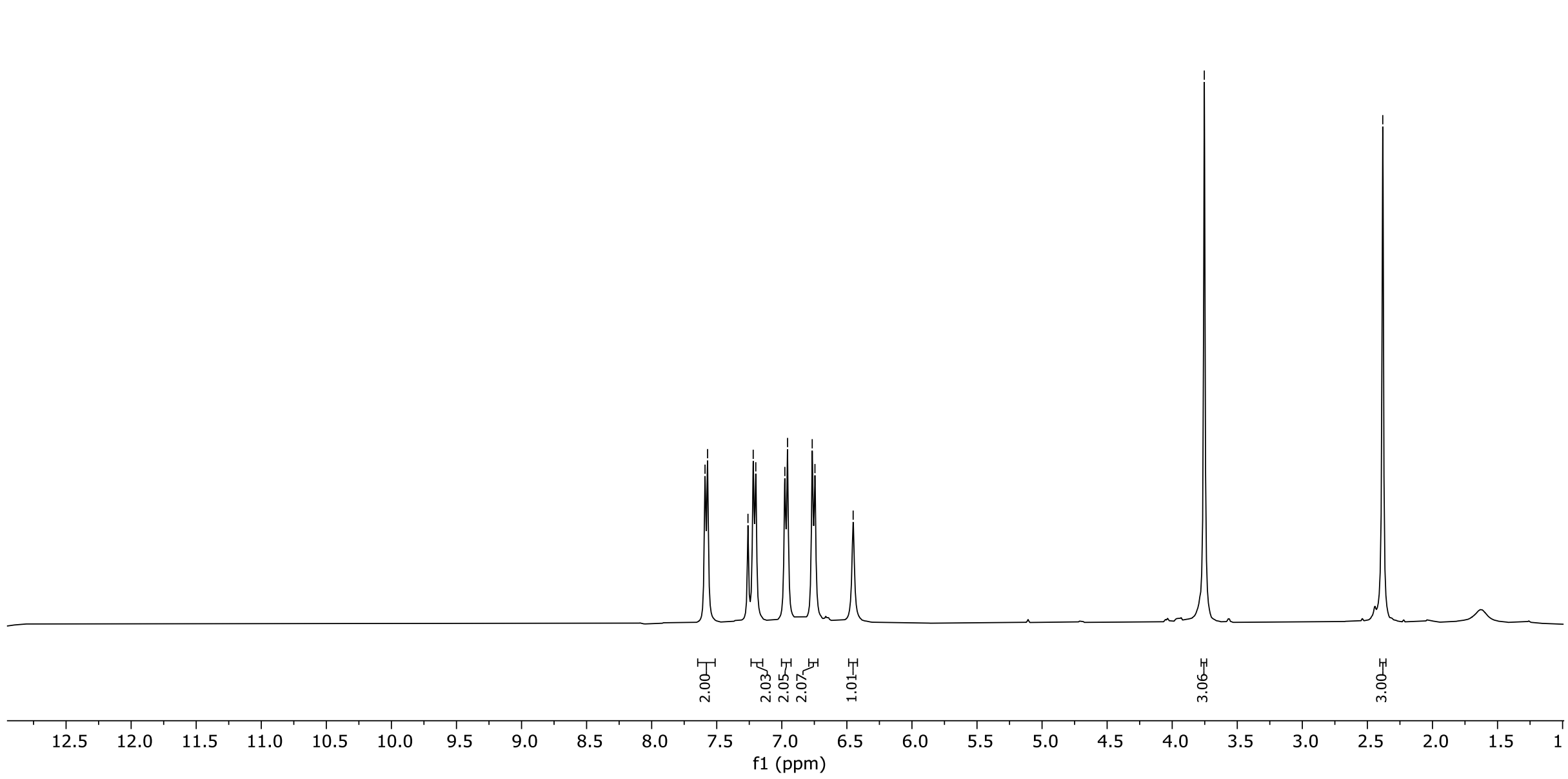

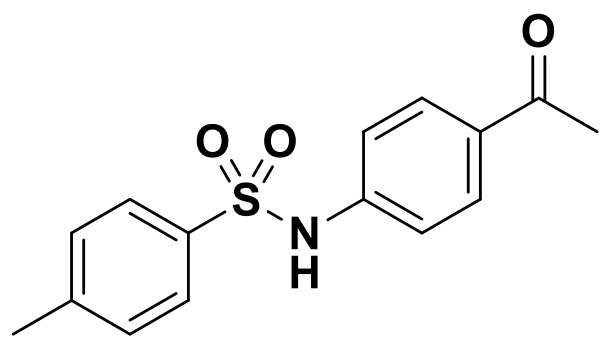

6

^{1}H NMR, 400 MHz, $CDCl_3$

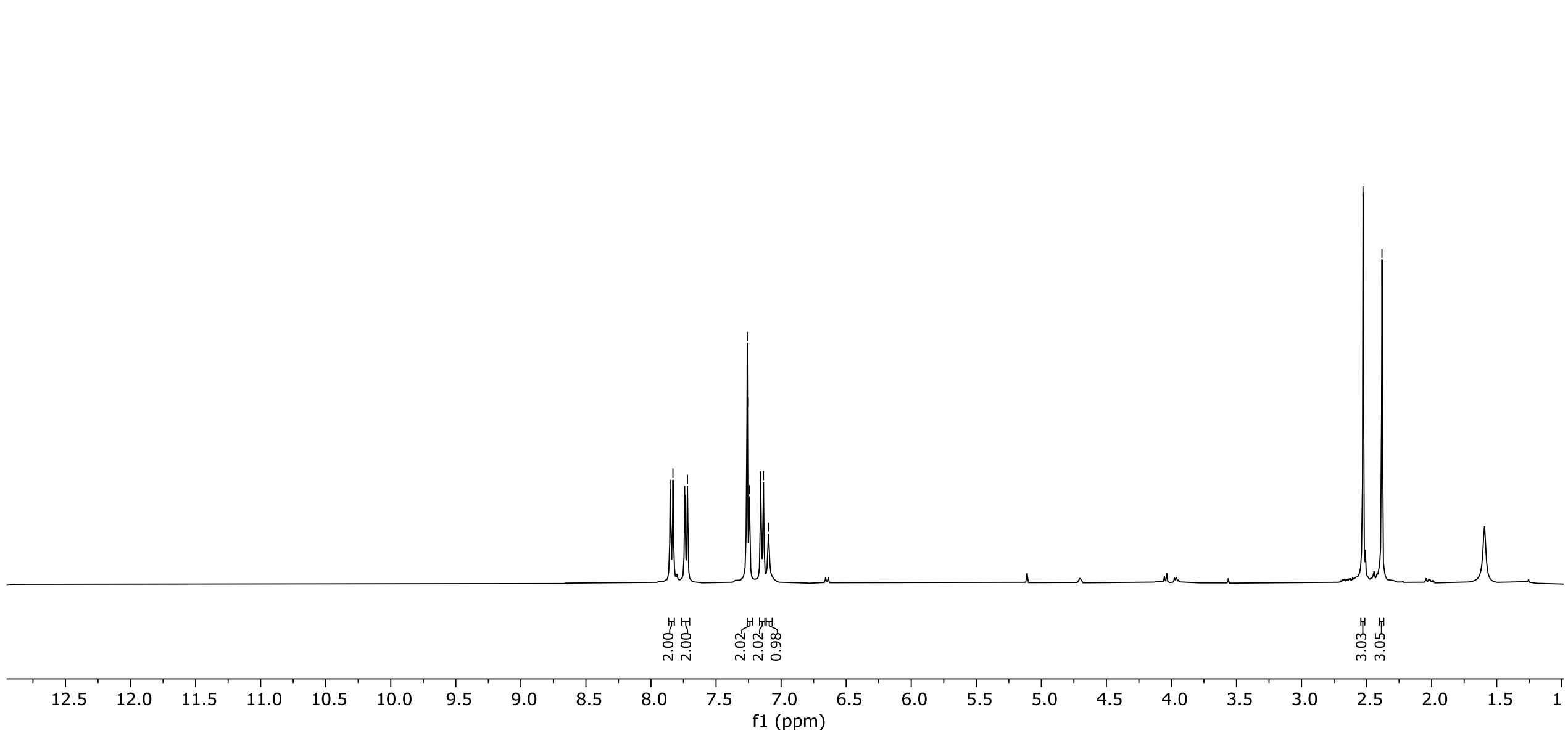

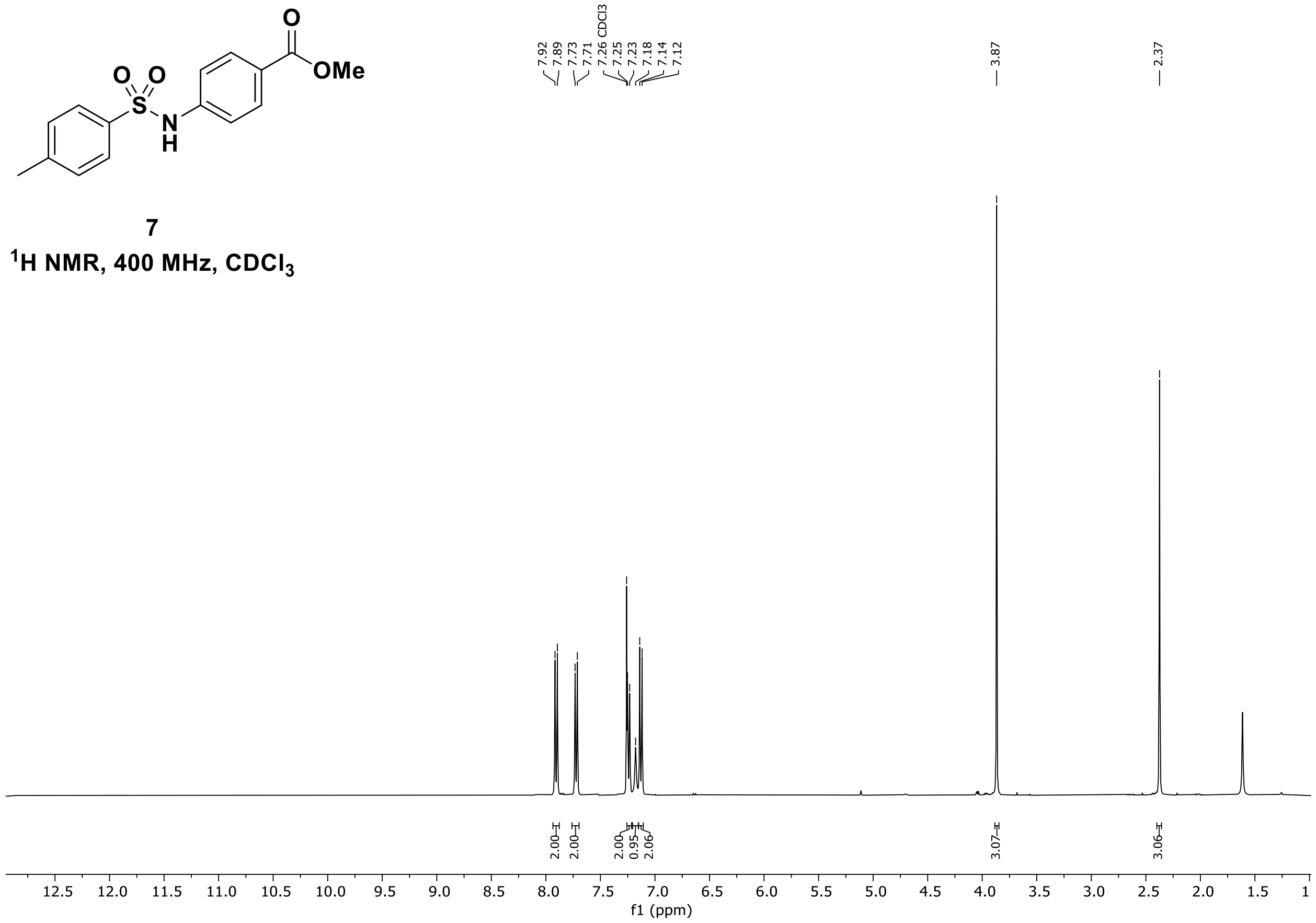

7

^{1}H NMR, 400 MHz, $CDCl_3$

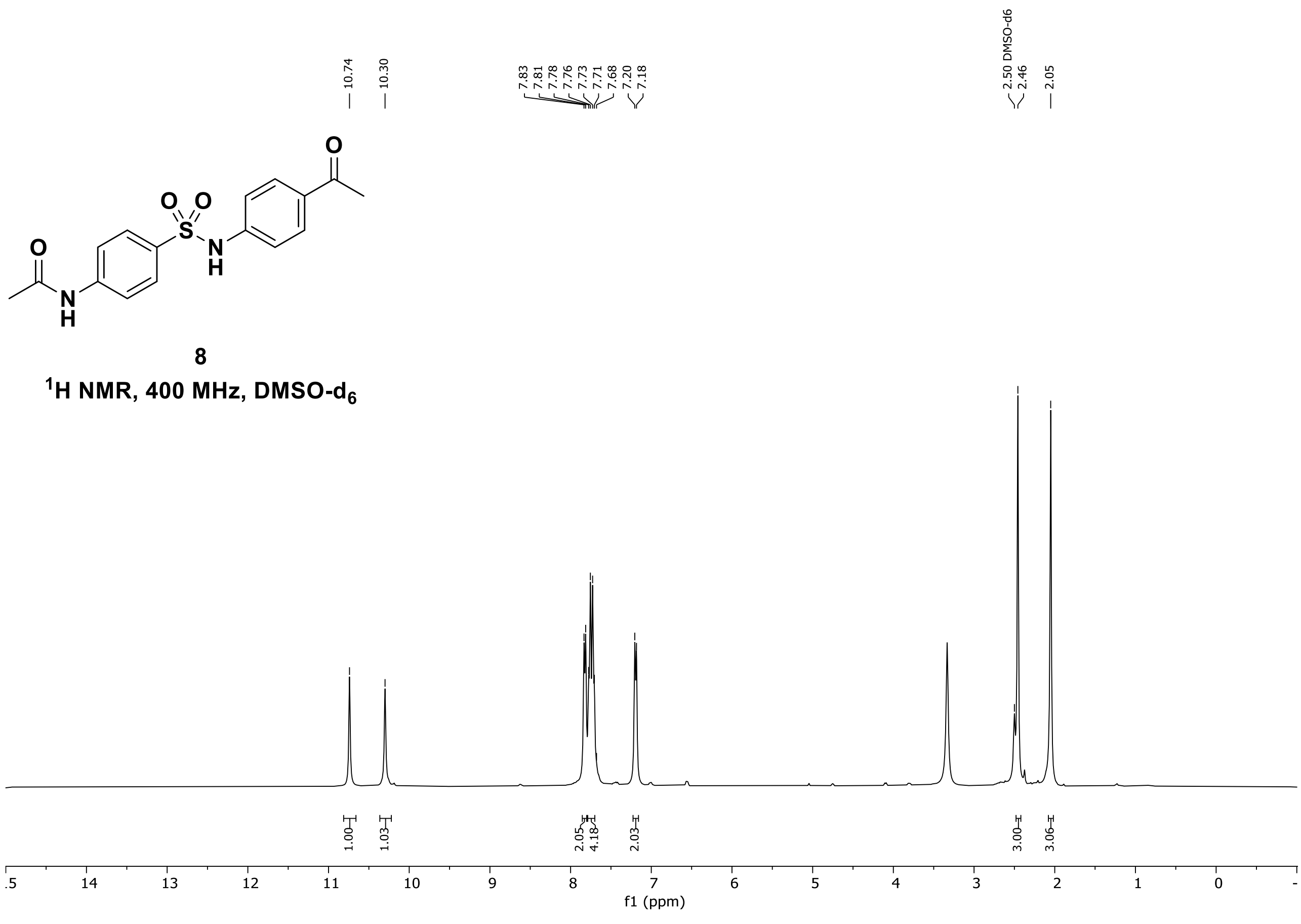

8

^{1}H NMR, 400 MHz, DMSO-d_6

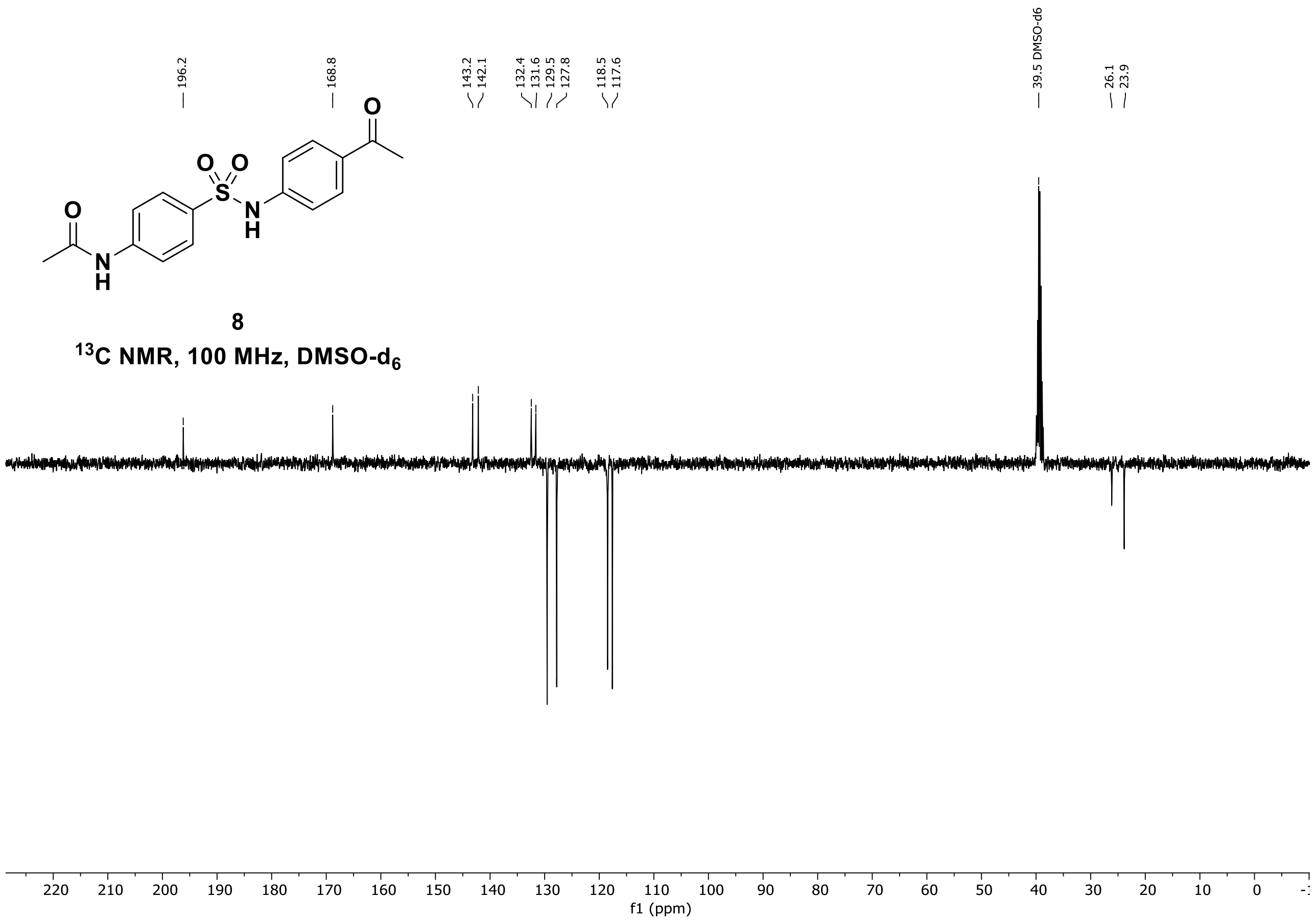

8
^{13}C NMR, 100 MHz, DMSO-d$_6$
196.2
168.8
143.2
142.1
132.4
131.6
129.5
127.8
118.5
117.6
39.5 DMSO-d6
26.1
23.9
220 210 200 190 180 170 160 150 140 130 120 110 100 90 80 70 60 50 40 30 20 10 0
f1 (ppm)

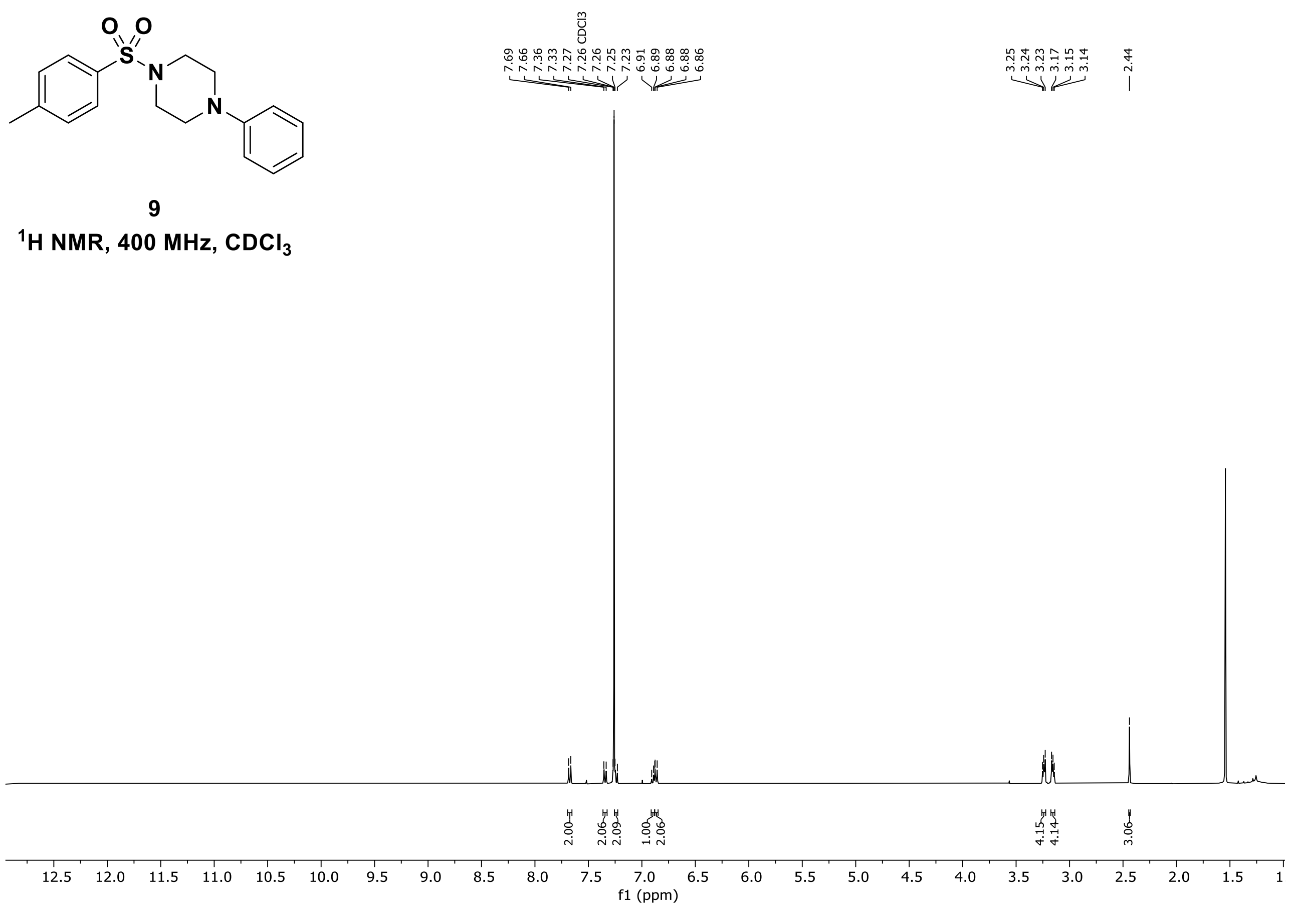

9
^{1}H NMR, 400 MHz, CDCl$_3$
7.69
7.66
7.36
7.33
7.27
7.26 CDCl3
7.26
7.25
7.23
6.91
6.89
6.88
6.88
6.86
3.25
3.24
3.23
3.17
3.15
3.14
2.44
2.00
2.06
2.09
1.00
2.06
4.15
4.14
3.06
12.5 12.0 11.5 11.0 10.5 10.0 9.5 9.0 8.5 8.0 7.5 7.0 6.5 6.0 5.5 5.0 4.5 4.0 3.5 3.0 2.5 2.0 1.5
f1 (ppm)

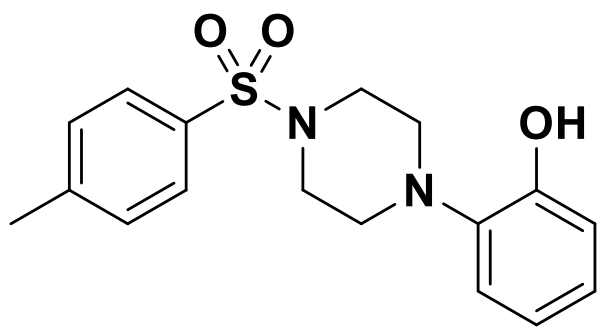

10

^{1}H NMR, 400 MHz, $CDCl_3$

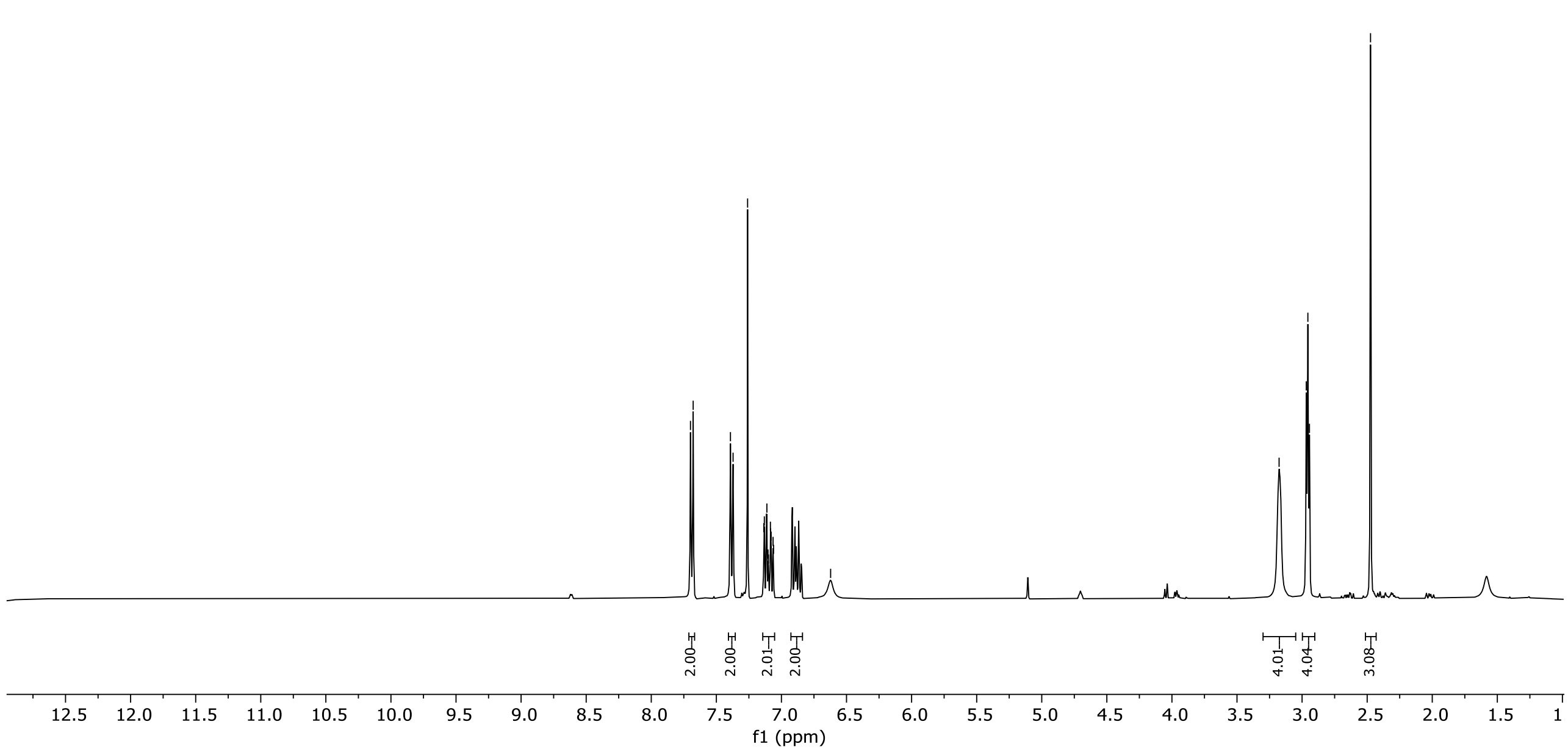

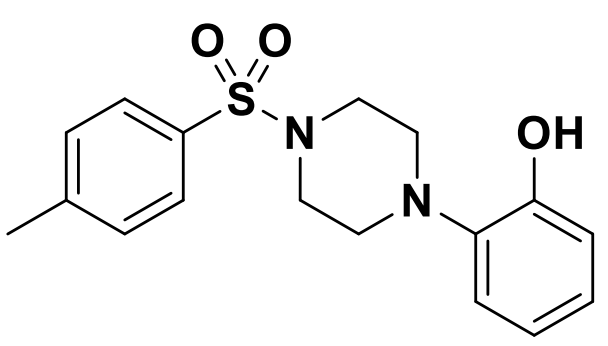

10

^{13}C NMR, 100 MHz, $CDCl_3$

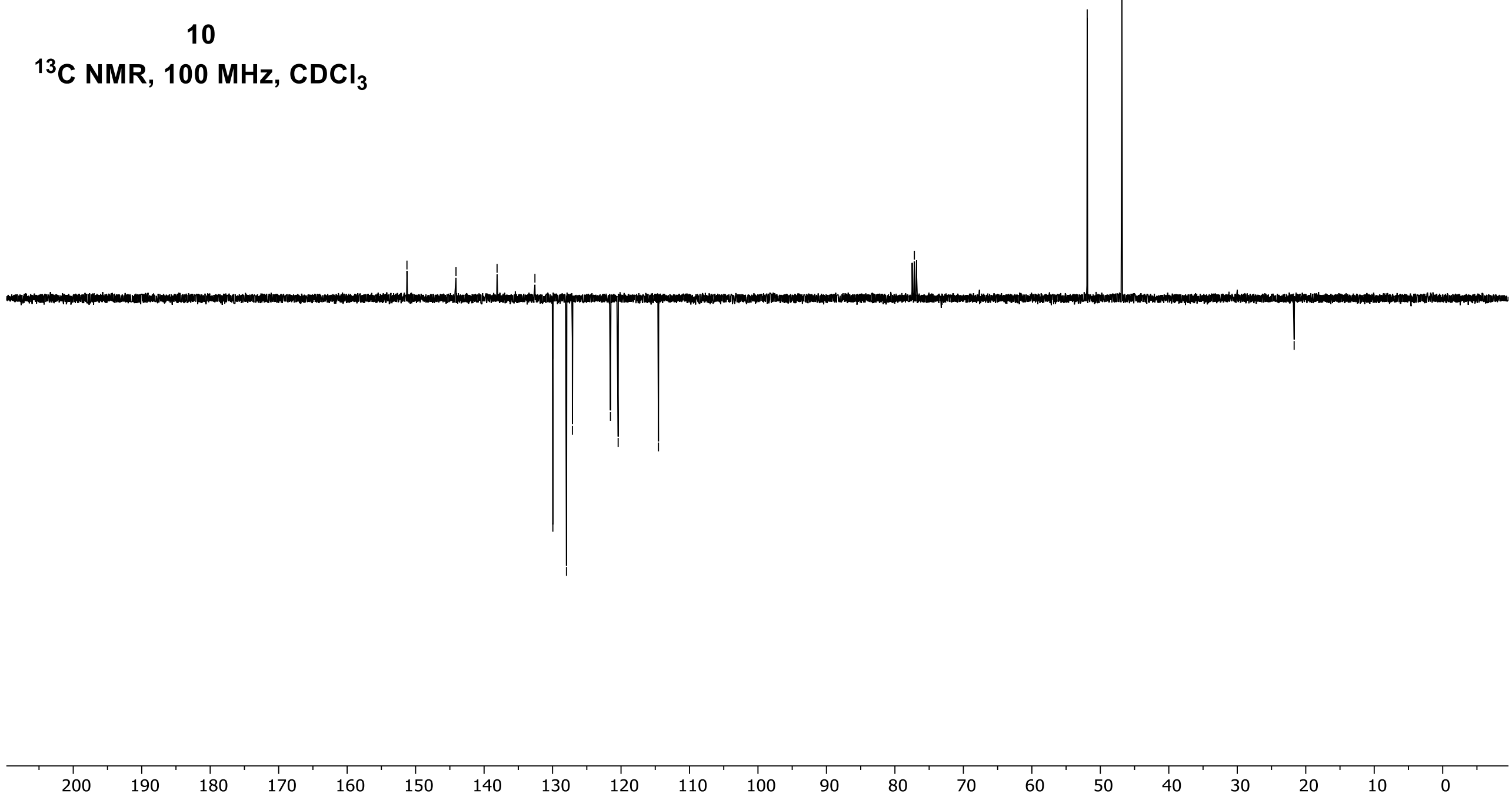

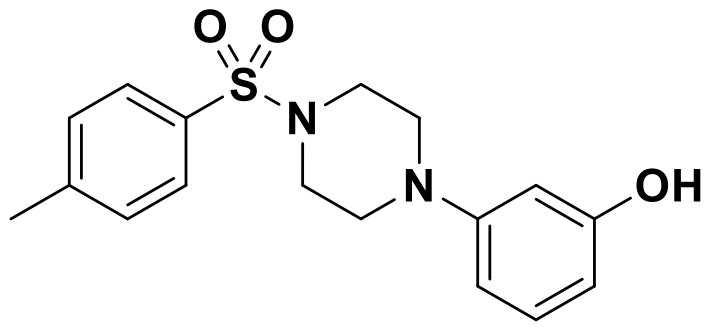

11

^{1}H NMR, 400 MHz, DMSO-d_6

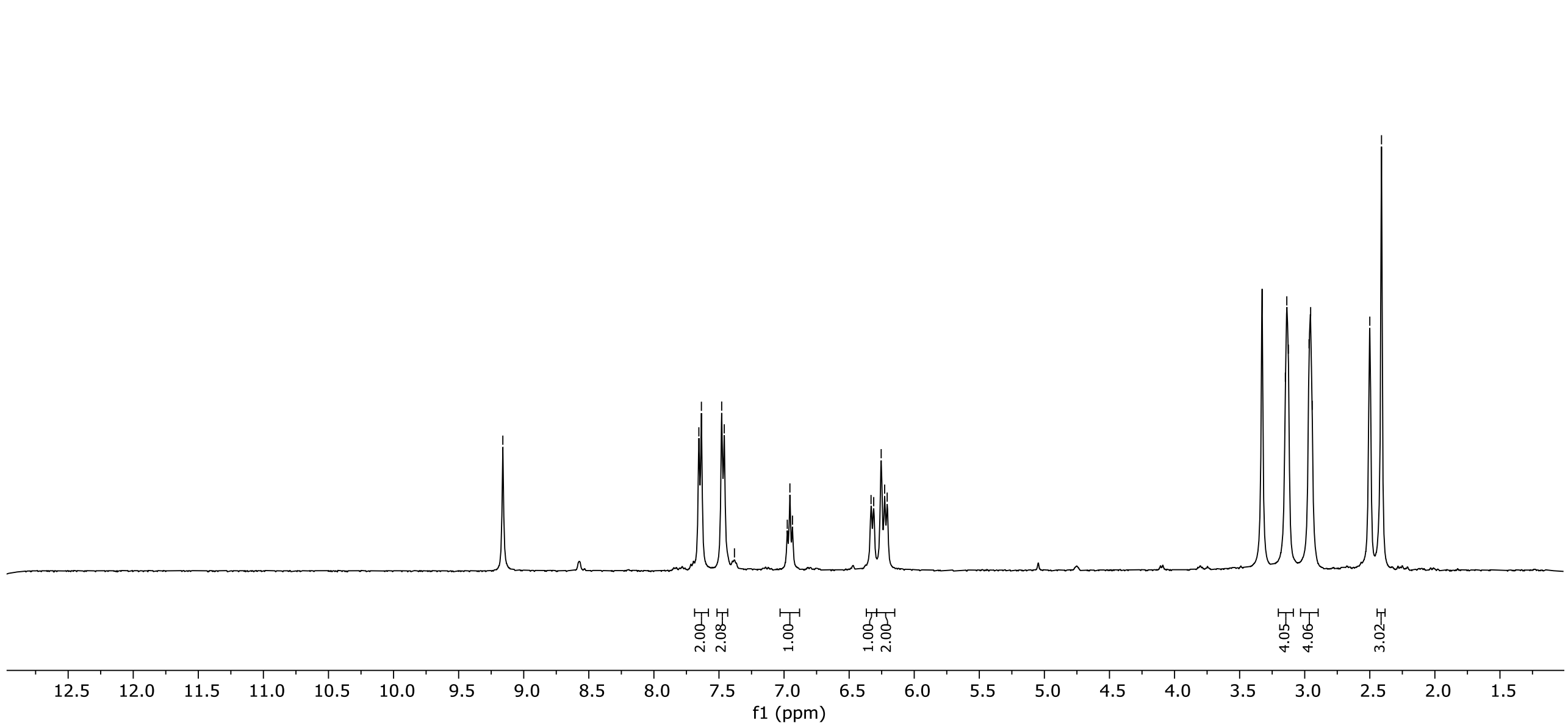

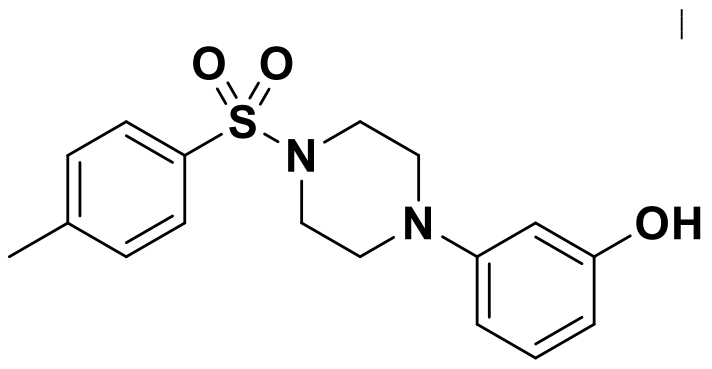

11

^{13}C NMR, 100 MHz, $CDCl_3$

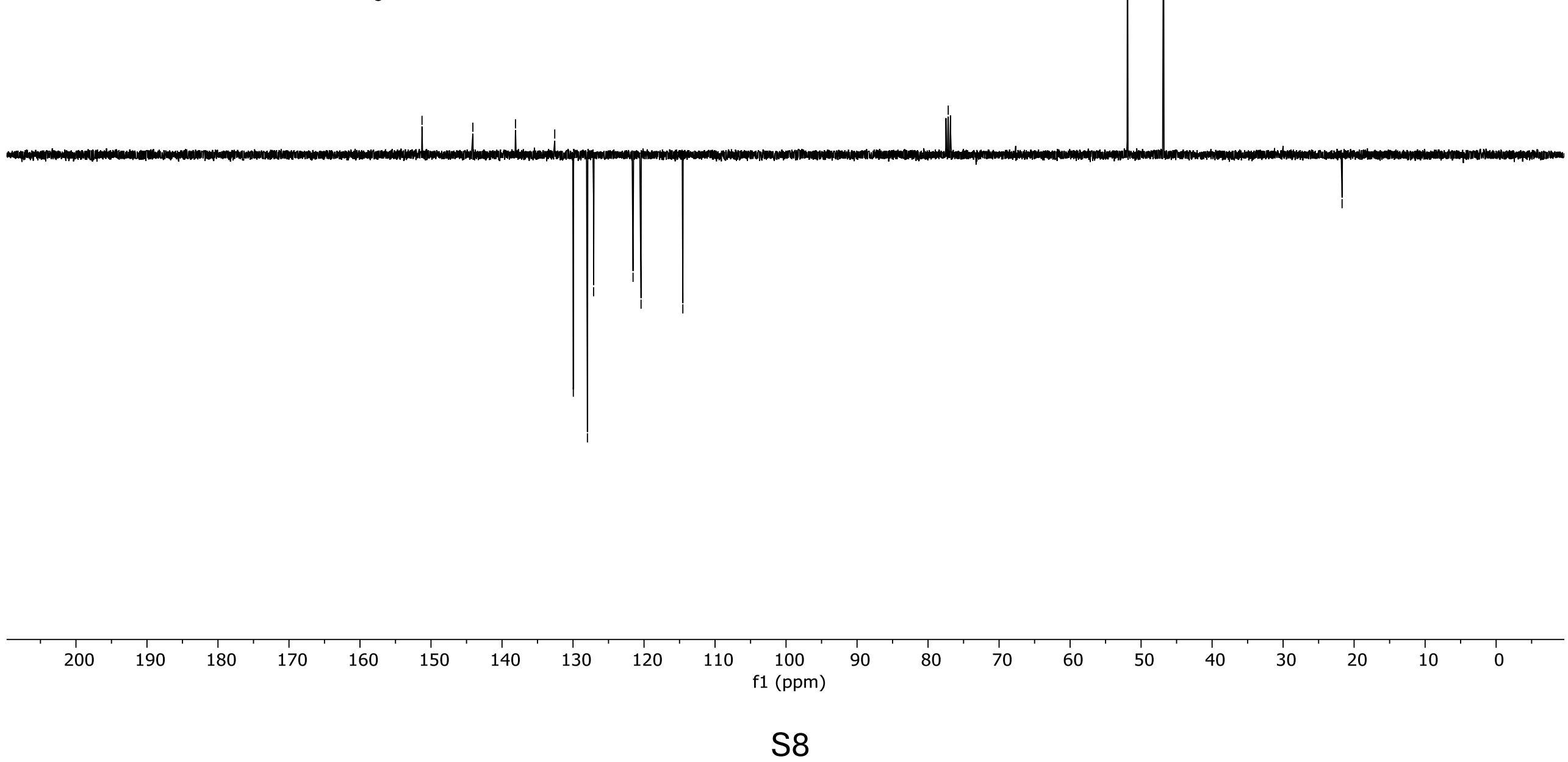

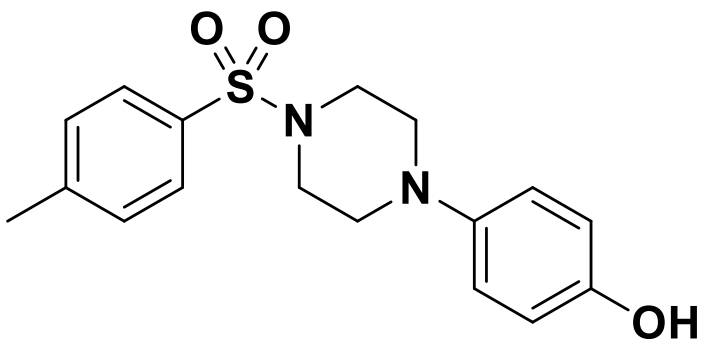

12

^{1}H NMR, 400 MHz, $CDCl_3$

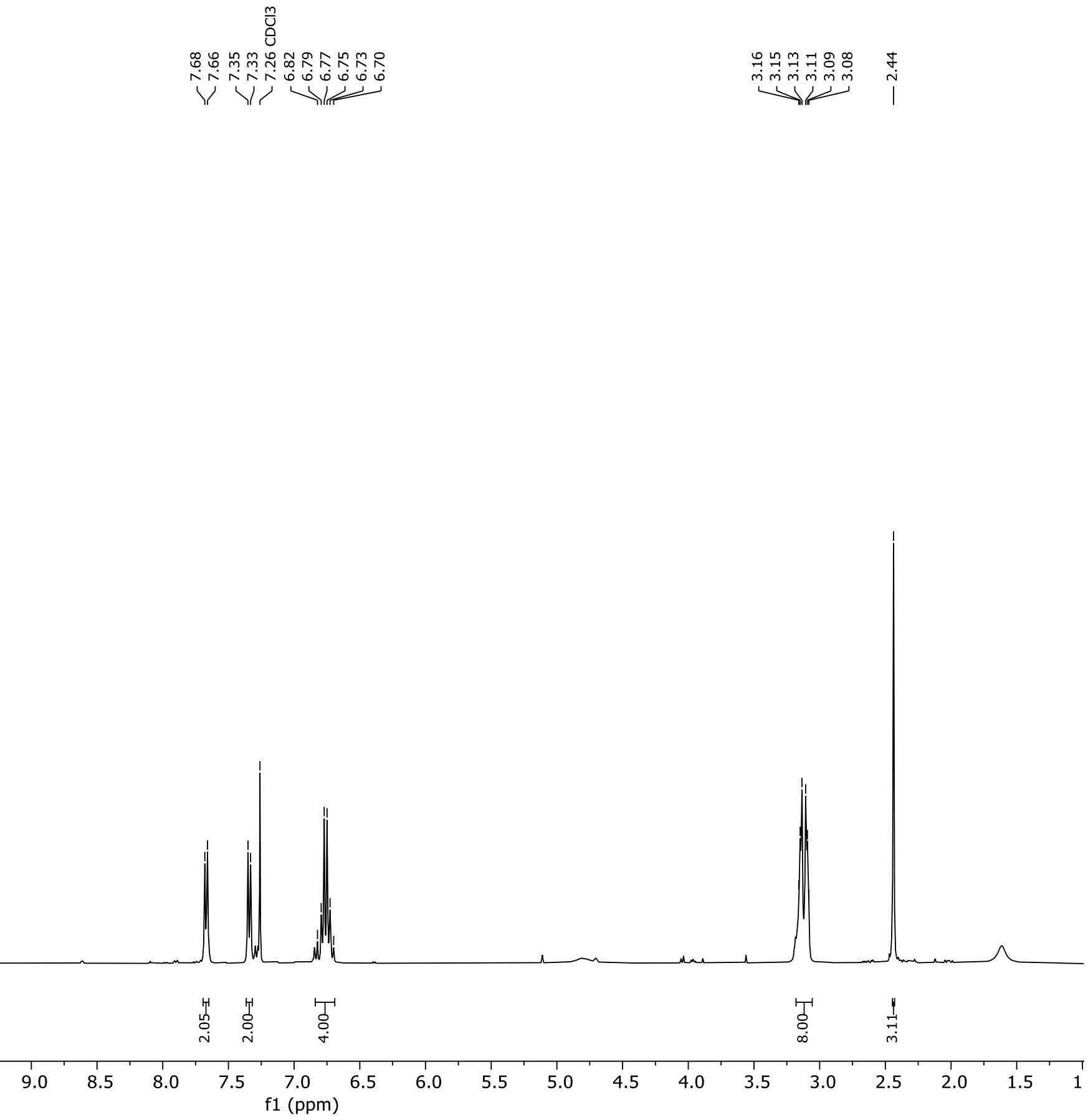

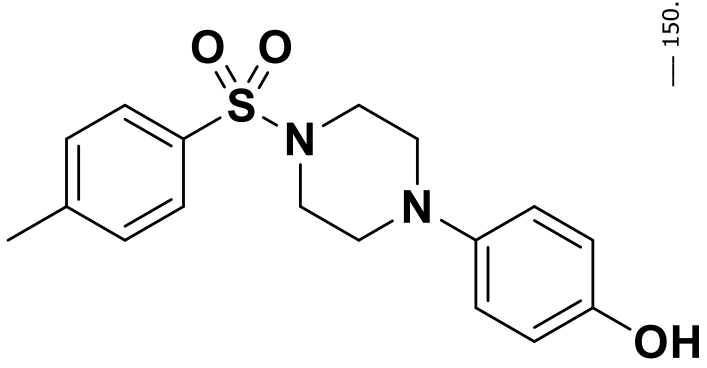

12

^{13}C NMR, 100 MHz, $CDCl_3$

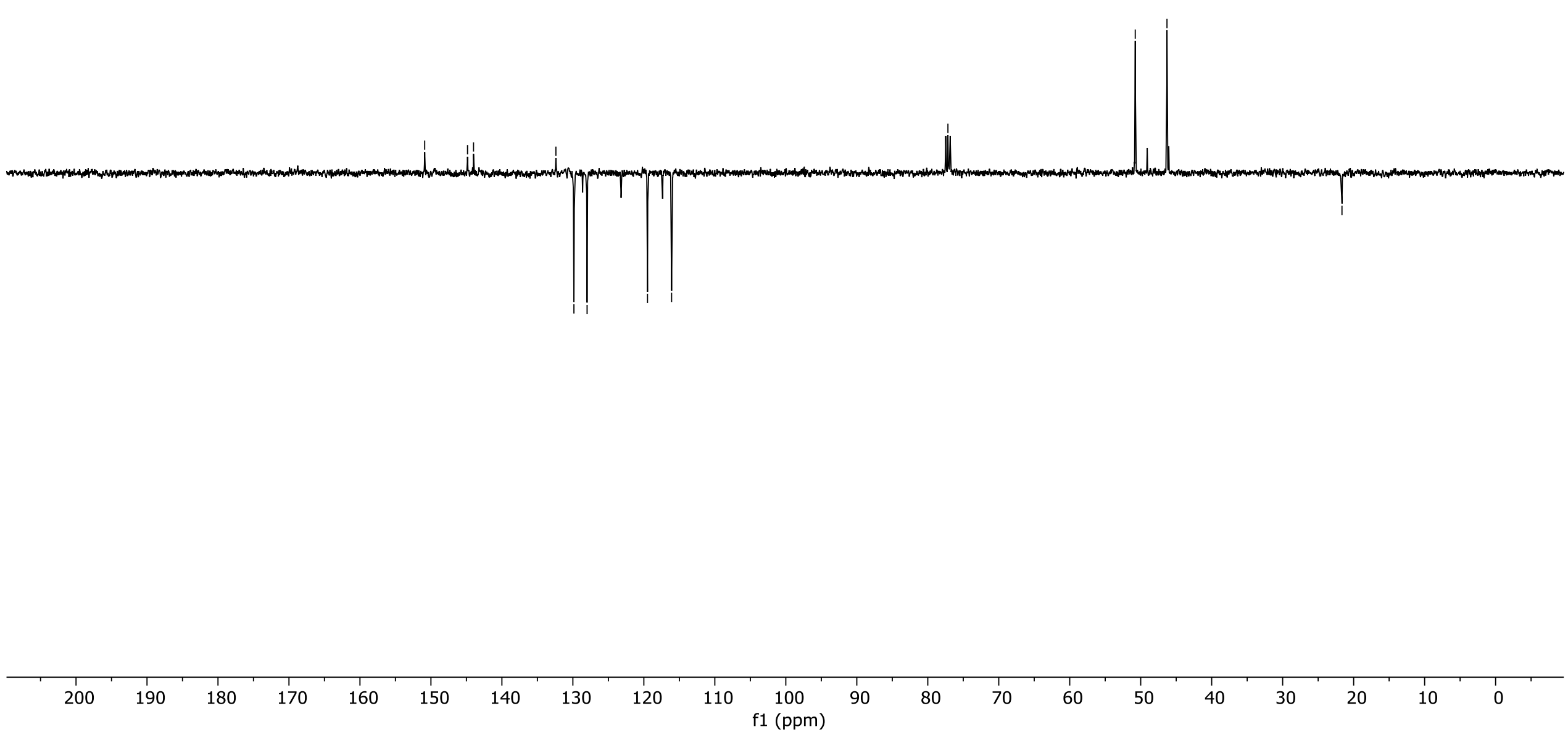

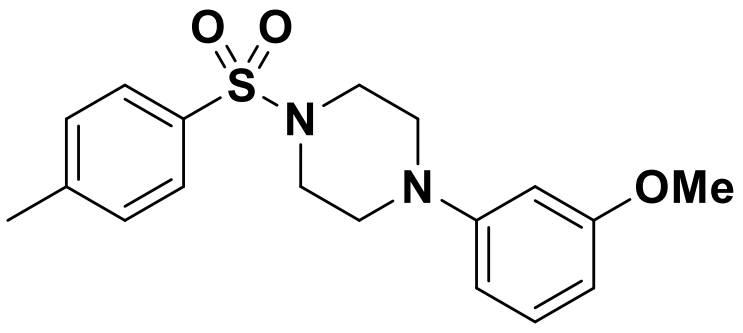

13

^{1}H NMR, 400 MHz, $CDCl_3$

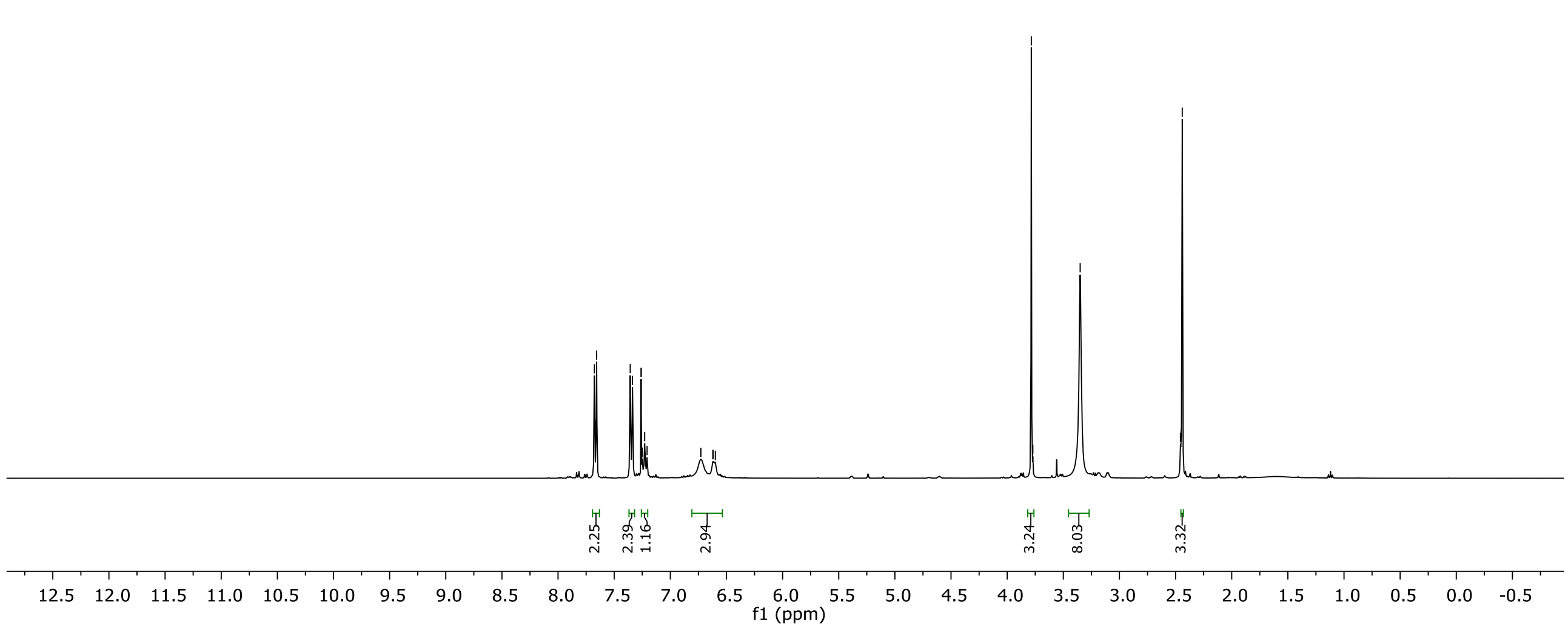

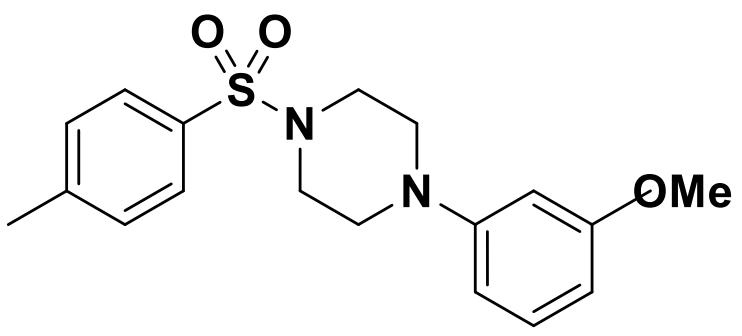

13

^{13}C NMR, 100 MHz, $CDCl_3$

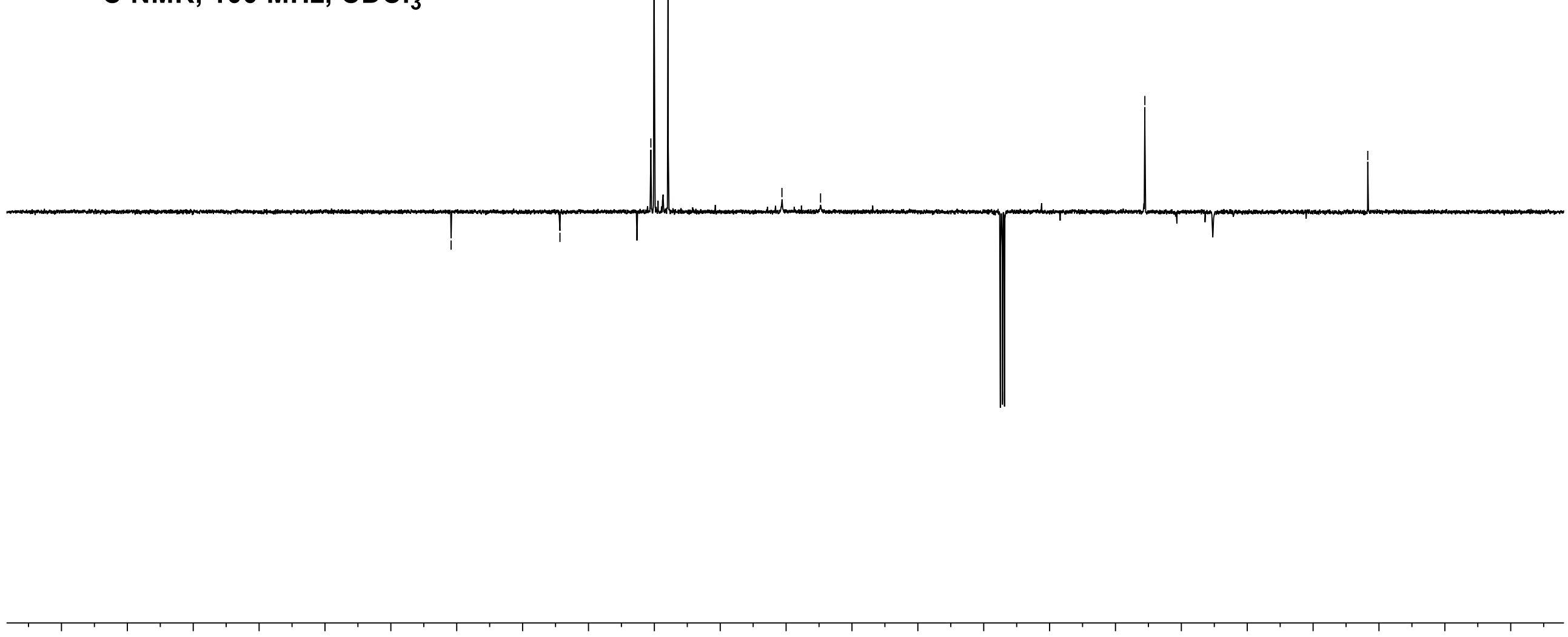

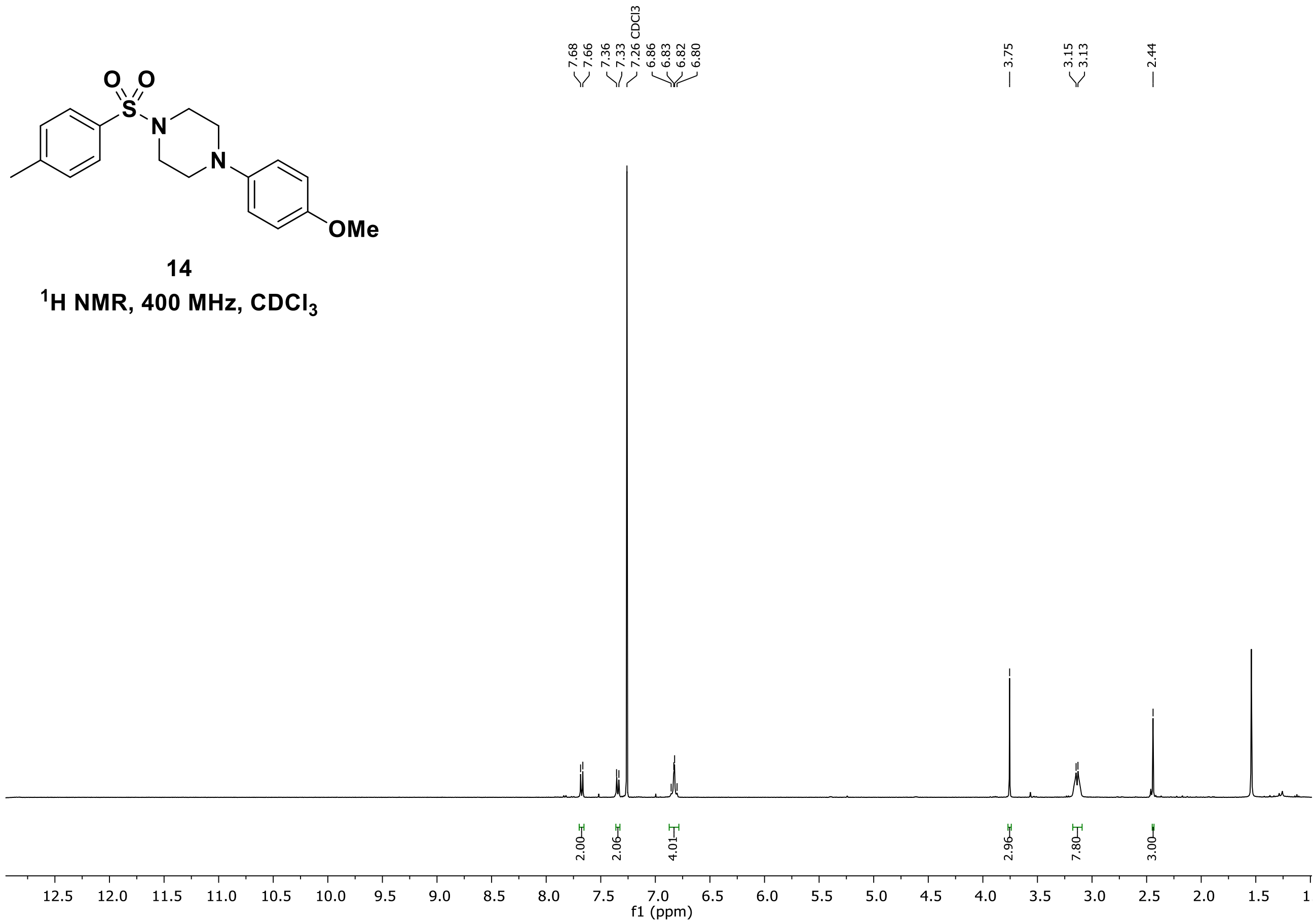

14

^{1}H NMR, 400 MHz, $CDCl_3$

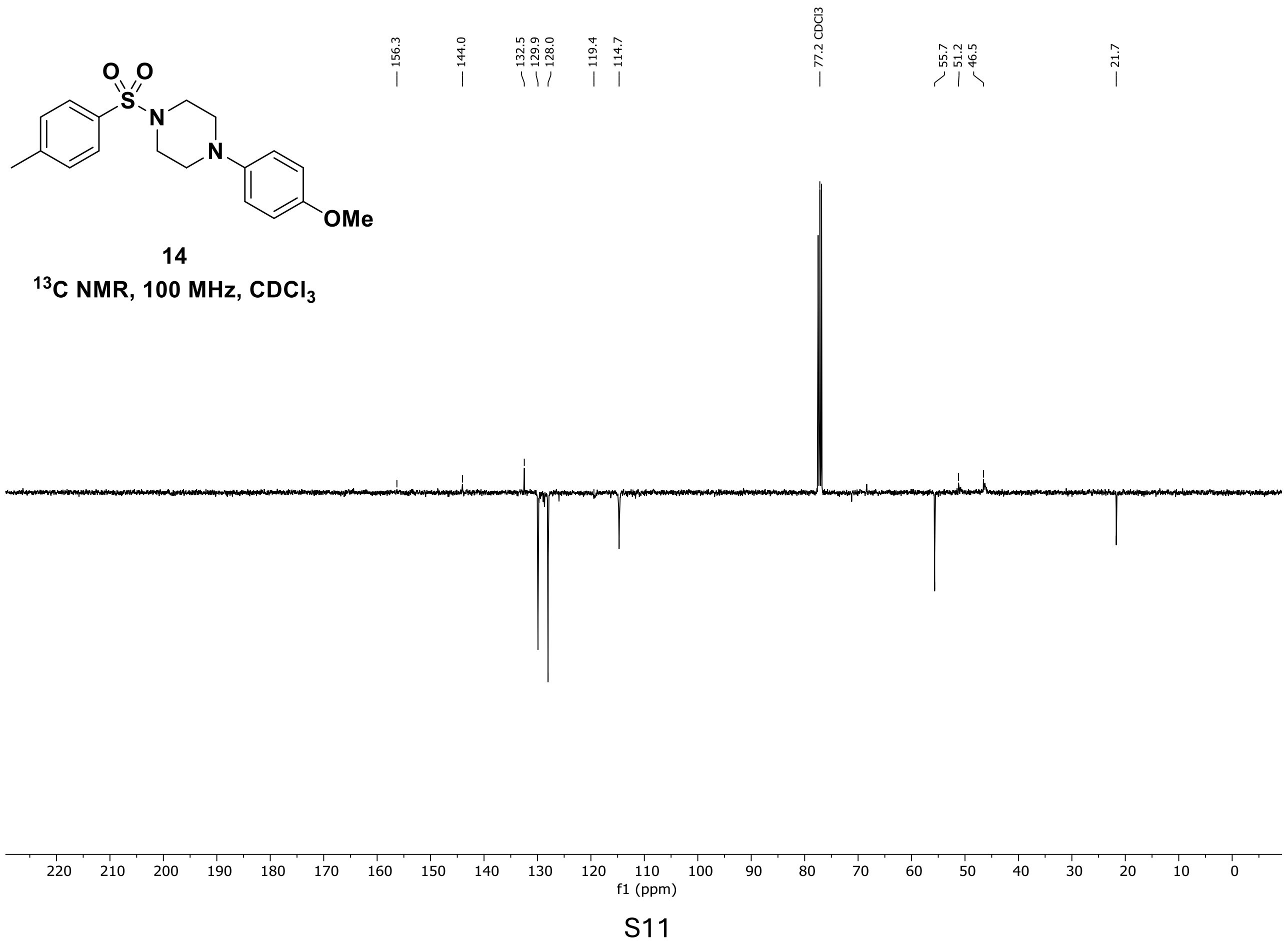

14

^{13}C NMR, 100 MHz, $CDCl_3$

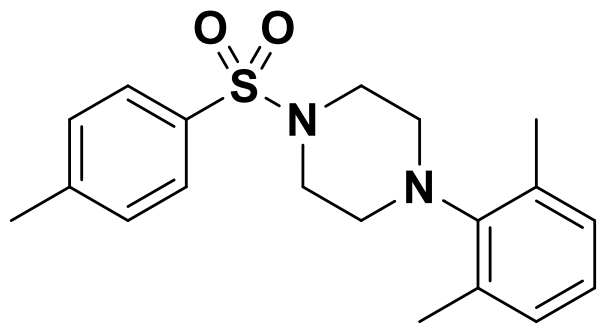

15

^{1}H NMR, 400 MHz, $CDCl_3$

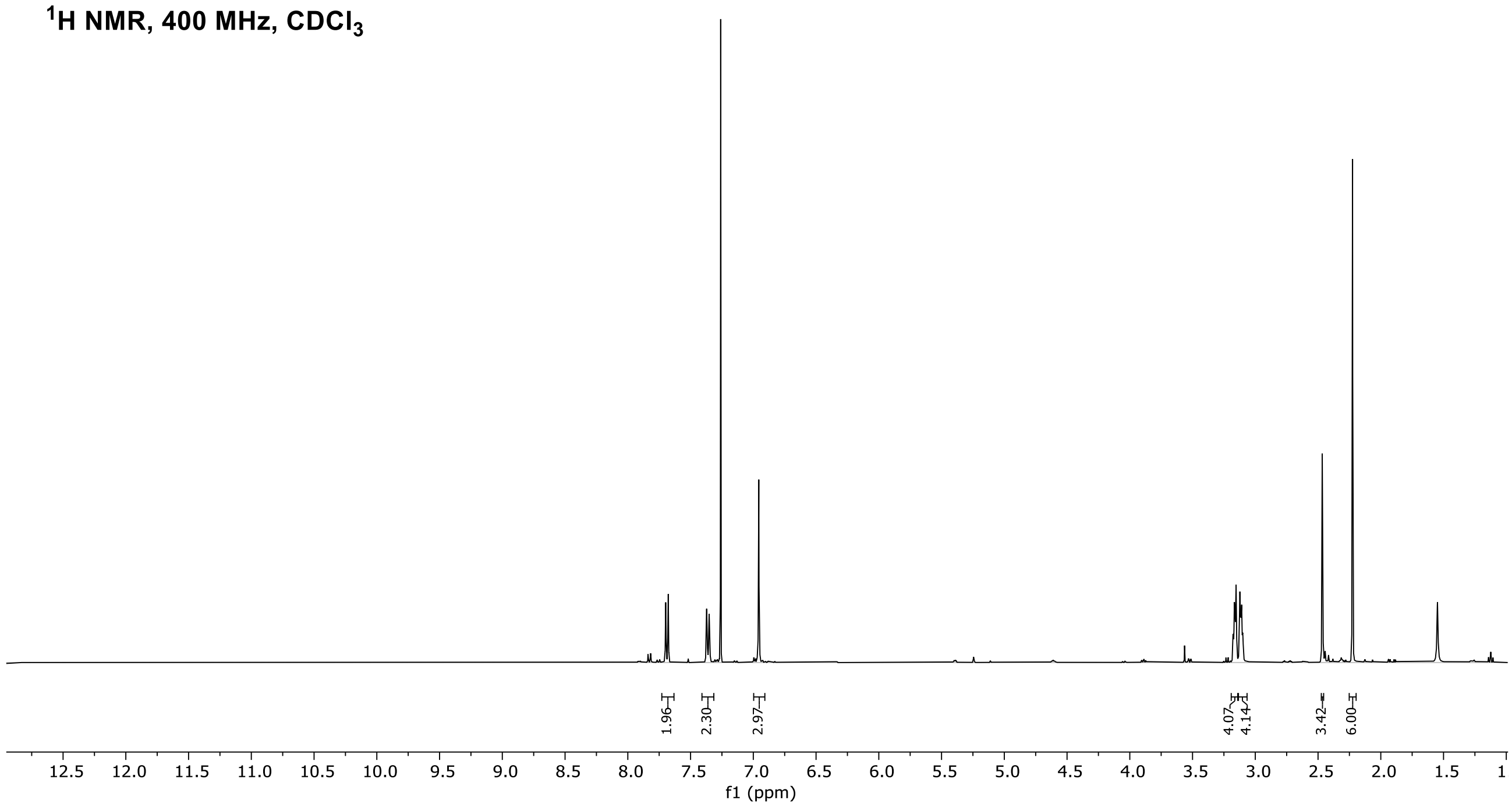

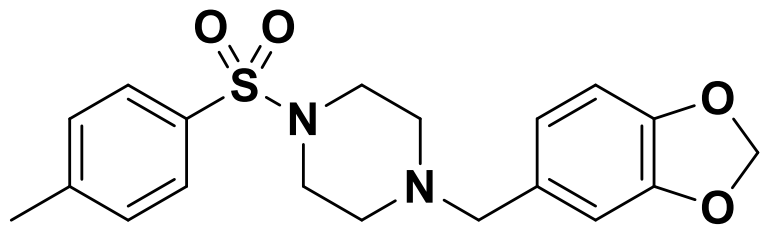

16

^{1}H NMR, 400 MHz, $CDCl_3$

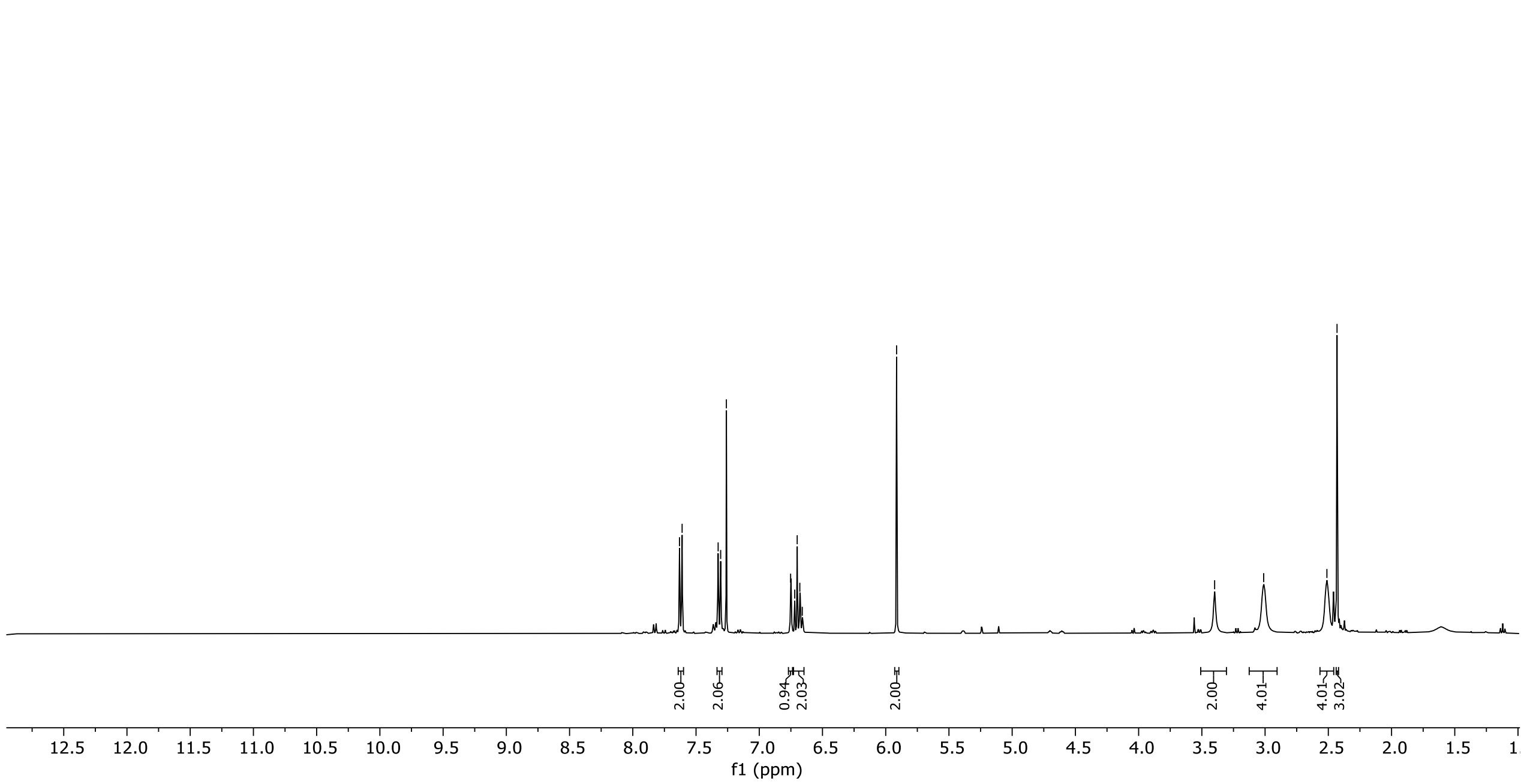

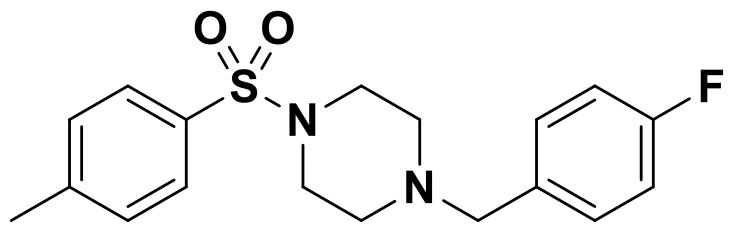

17
^{1}H NMR, 400 MHz, $CDCl_3$

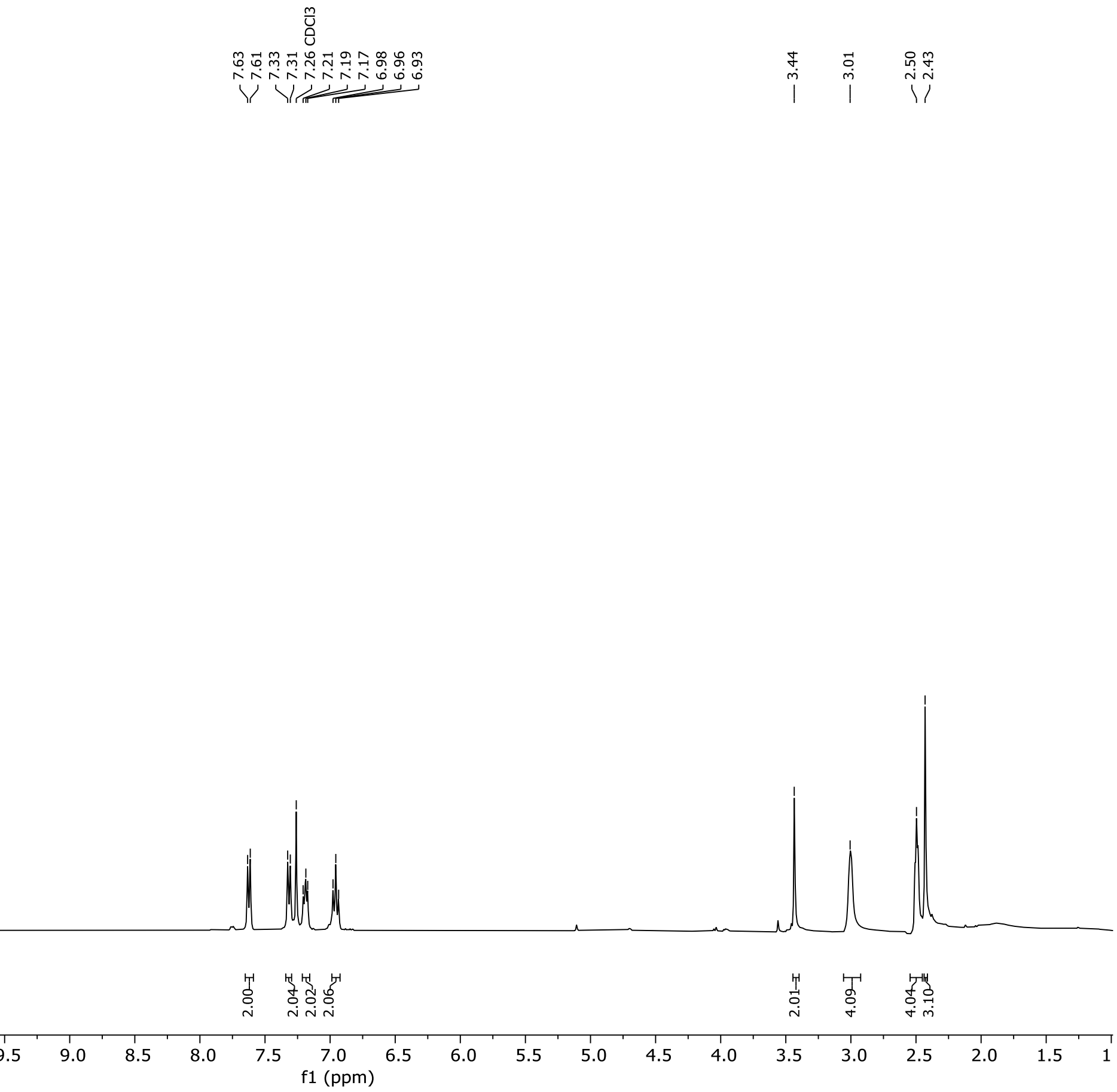

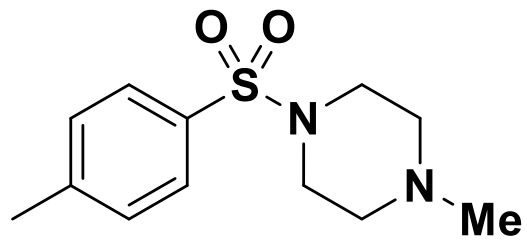

18
^{1}H NMR, 400 MHz, $CDCl_3$

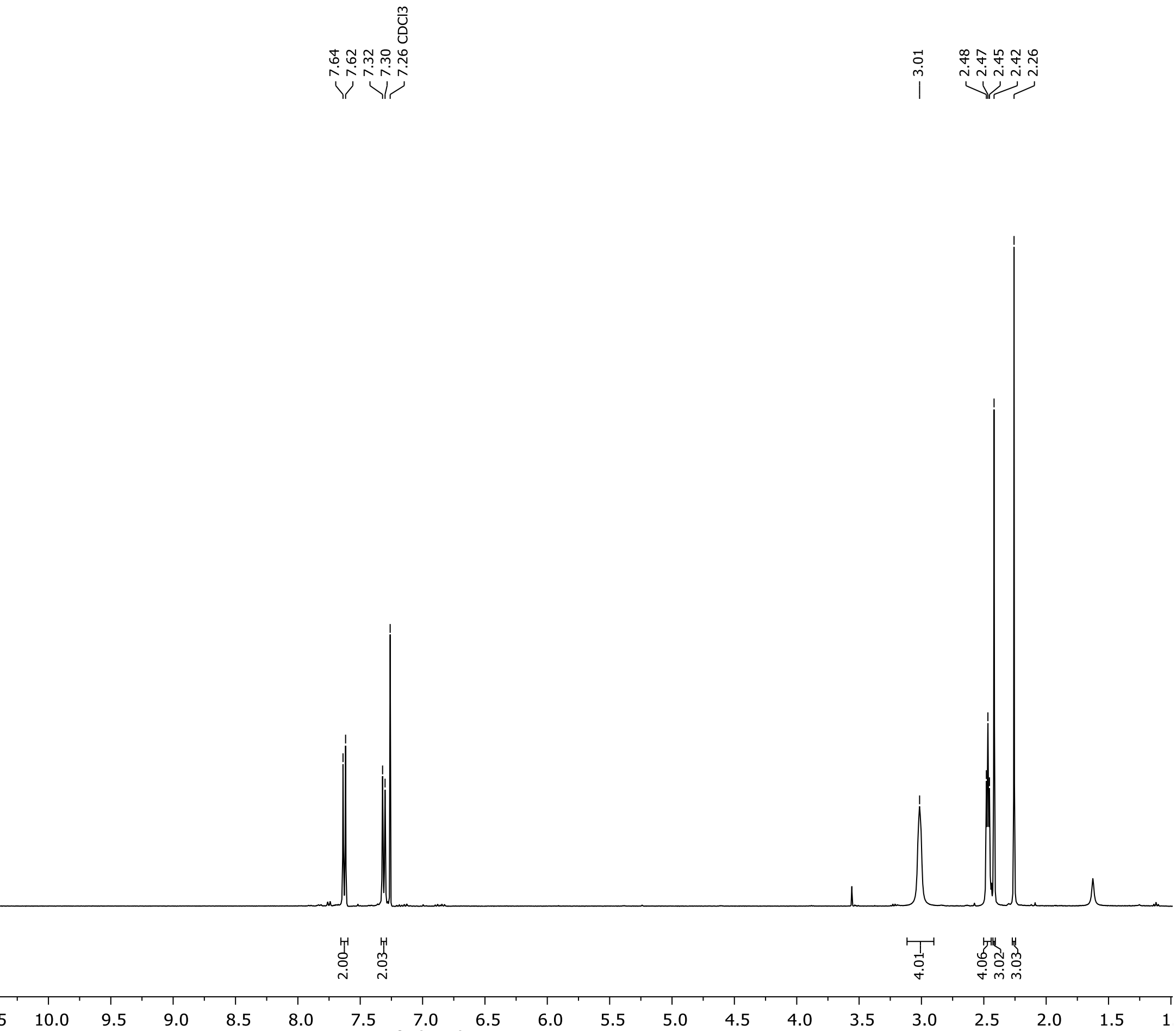

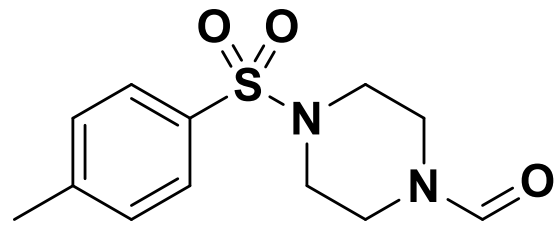

19
^{1}H NMR, 400 MHz, $CDCl_3$

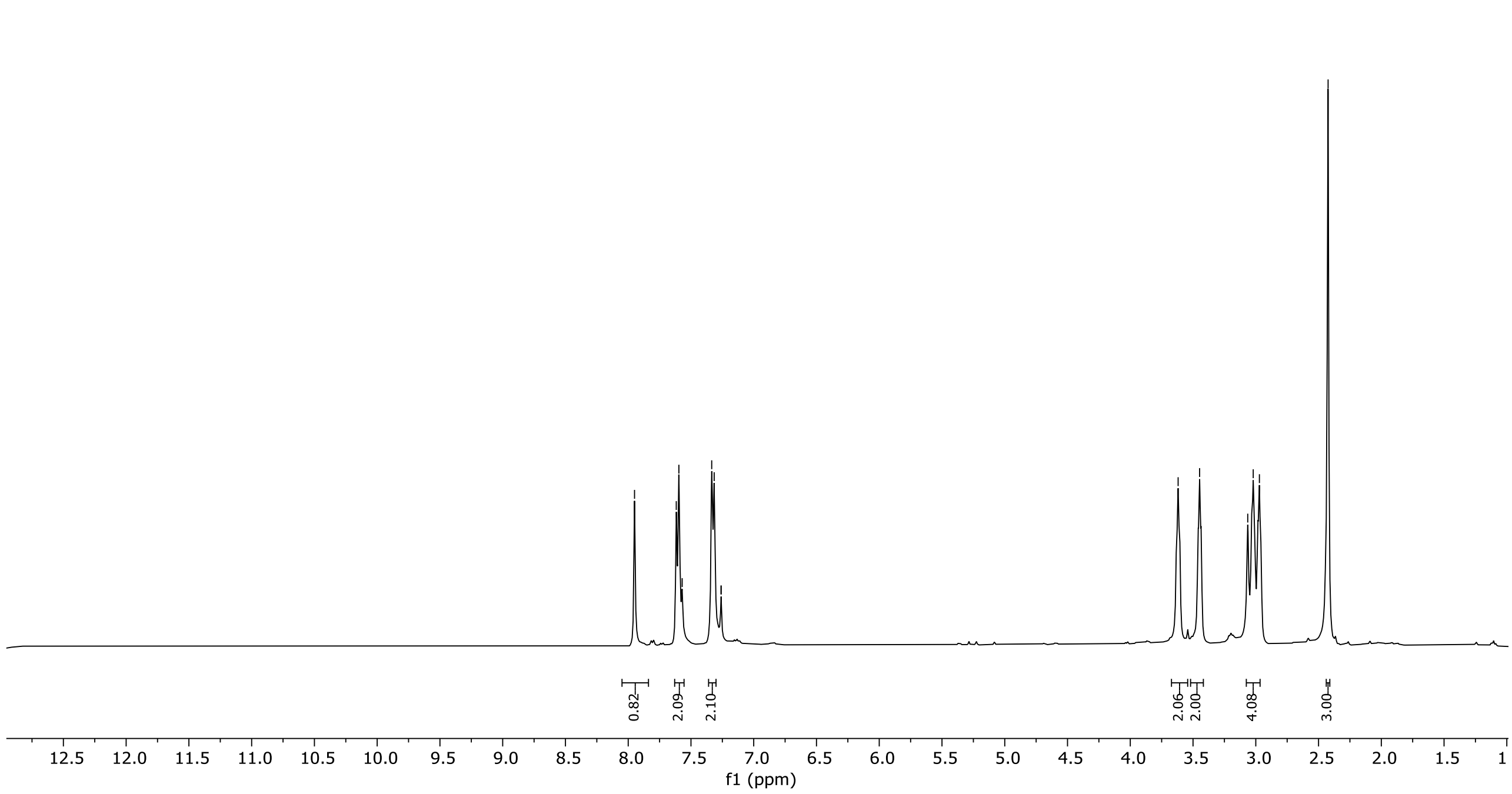

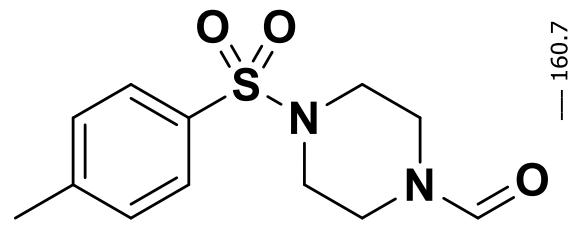

19
^{13}C NMR, 100 MHz, $CDCl_3$

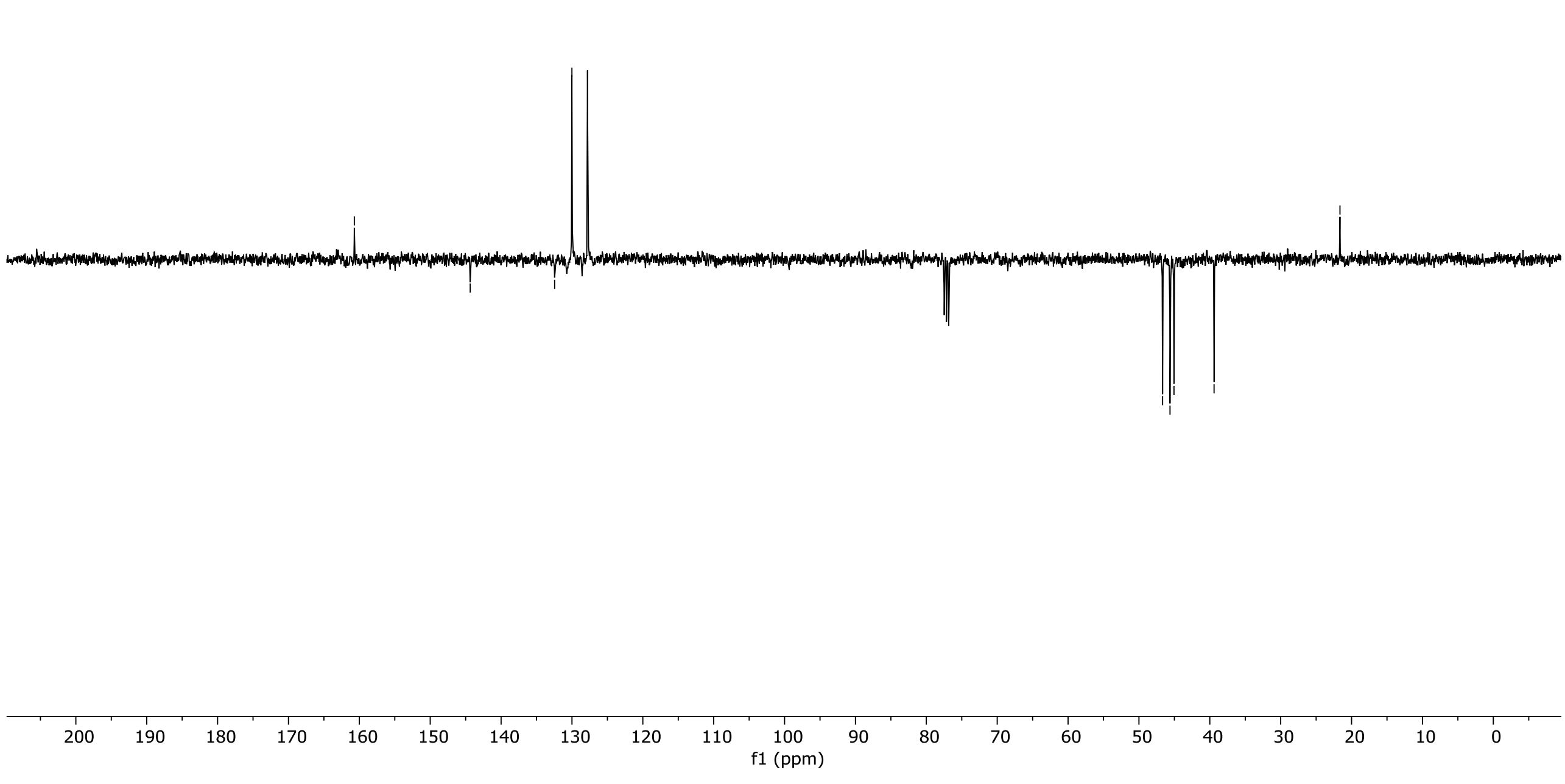

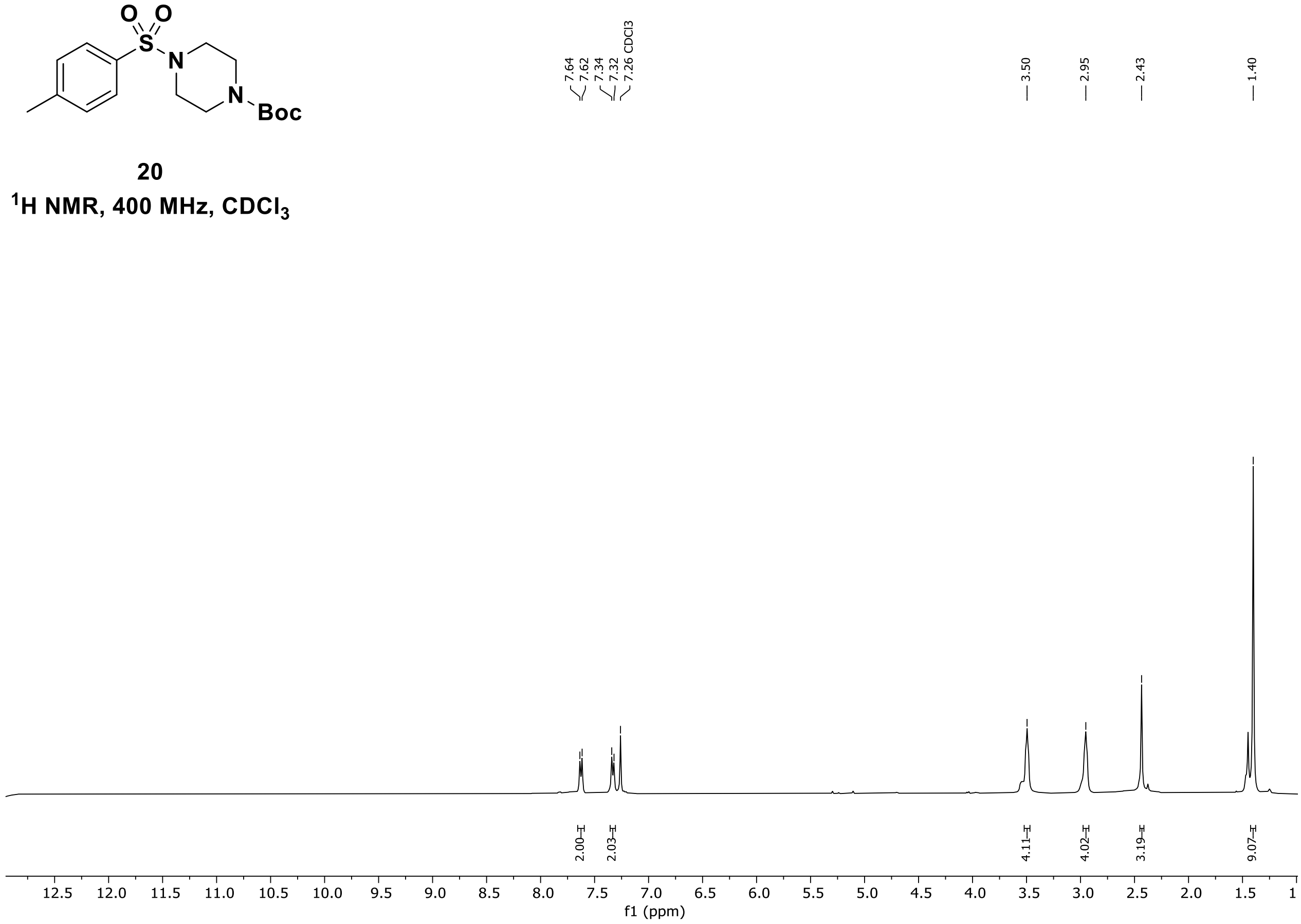

20

^{1}H NMR, 400 MHz, $CDCl_3$

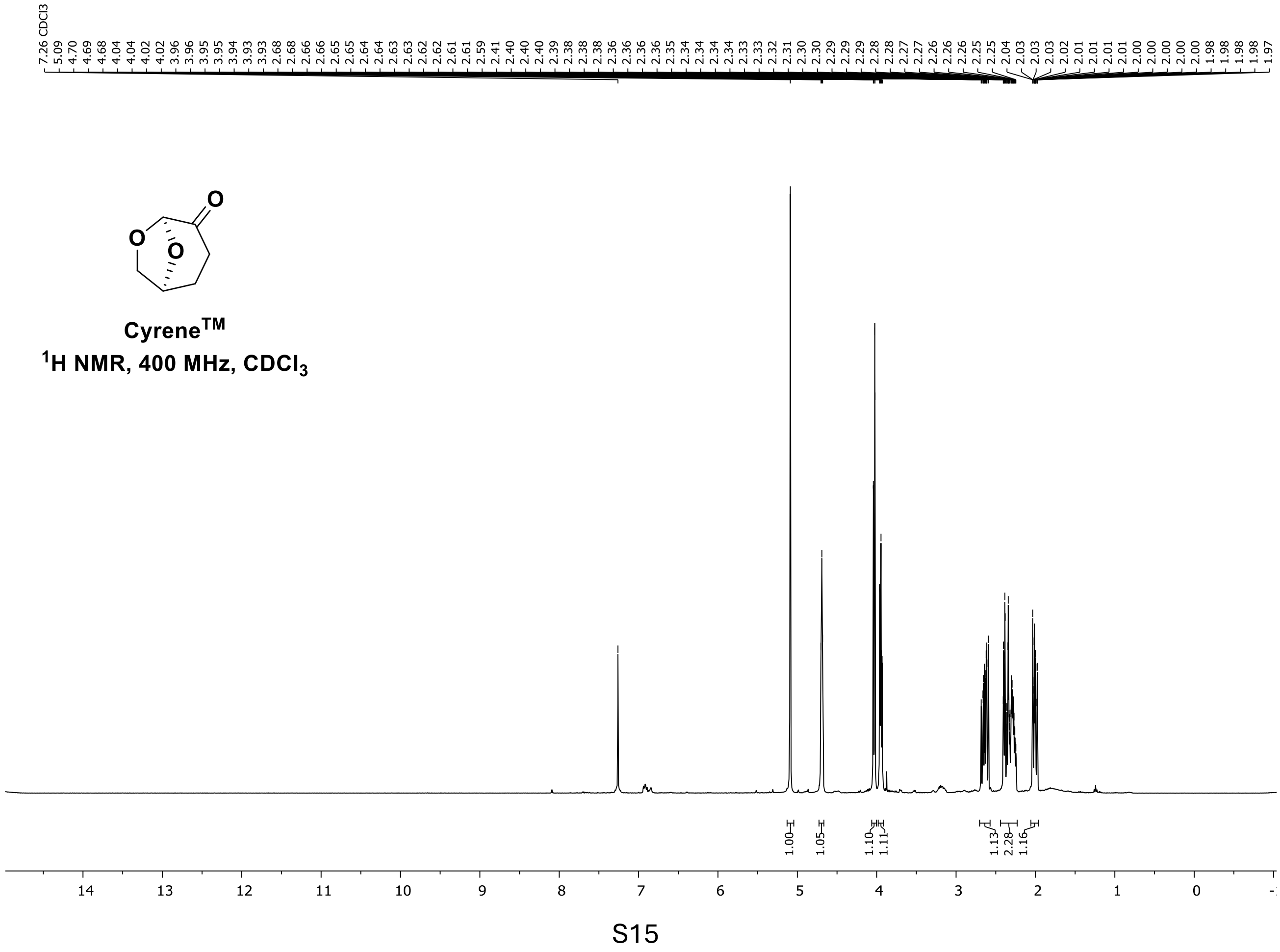

Cyrene^{TM}

^{1}H NMR, 400 MHz, $CDCl_3$
